# Finding motifs using DNA images derived from sparse representations

**DOI:** 10.1093/bioinformatics/btad378

**Published:** 2023-06-09

**Authors:** Shane K Chu, Gary D Stormo

**Affiliations:** Department of Computer Science and Engineering, Washington University in St. Louis, St. Louis, MO 63130, United States; Department of Genetics, Washington University School of Medicine, St. Louis, MO 63110, United States

## Abstract

**Motivation:**

Motifs play a crucial role in computational biology, as they provide valuable information about the binding specificity of proteins. However, conventional motif discovery methods typically rely on simple combinatoric or probabilistic approaches, which can be biased by heuristics such as substring-masking for multiple motif discovery. In recent years, deep neural networks have become increasingly popular for motif discovery, as they are capable of capturing complex patterns in data. Nonetheless, inferring motifs from neural networks remains a challenging problem, both from a modeling and computational standpoint, despite the success of these networks in supervised learning tasks.

**Results:**

We present a principled representation learning approach based on a hierarchical sparse representation for motif discovery. Our method effectively discovers gapped, long, or overlapping motifs that we show to commonly exist in next-generation sequencing datasets, in addition to the short and enriched primary binding sites. Our model is fully interpretable, fast, and capable of capturing motifs in a large number of DNA strings. A key concept emerged from our approach—enumerating at the image level—effectively overcomes the k-mers paradigm, enabling modest computational resources for capturing the long and varied but conserved patterns, in addition to capturing the primary binding sites.

**Availability and implementation:**

Our method is available as a Julia package under the MIT license at https://github.com/kchu25/MOTIFs.jl, and the results on experimental data can be found at https://zenodo.org/record/7783033.

## 1 Introduction

Identifying conserved substrings in a set of unaligned DNA strings is a fundamental challenge in computational biology. These conserved substrings, known as motifs, emerge due to evolutionary forces, and are known to play a crucial role in regulatory mechanisms. Elucidating the regulatory motifs present in specific genomic regions, such as the promoters and enhancers, can shed light on gene regulation mechanisms and contribute to our understanding of biological processes. As such, accurately identifying motifs is an essential step toward understanding the complex interplay between genes and their regulation.

Motifs are often inferred from the representations of computational models applied to DNA strings. Traditionally, motifs are inferred directly, as the conventional methods typically depict the motifs as consensus strings (Li *et al.* 1999), product multinomials (Bailey and Elkan 1995, Liu *et al.* 1995), or position weight matrices (Hertz and Stormo 1999, Heinz *et al.* 2010, Bailey 2021). While the traditional methods, e.g. STREME and HOMER, are efficient in identifying the primary motif, they often employ heuristics such as substring masking that turns the methodology into a sequential procedure. This methodological approach can make it challenging to discover secondary motifs in the dataset in a principled manner, as secondary motifs usually share identical patterns with the primary motifs. Moreover, these conventional methods frequently rely on k-mer enumeration to generate initial seeds for the optimization subroutine, constraining them to identify only ungapped motifs and limiting the maximum length of motifs that can be discovered. For example, STREME version 5.5.1 by default can only detect motifs that are up to 30 base pairs in length.

**Figure 1.**
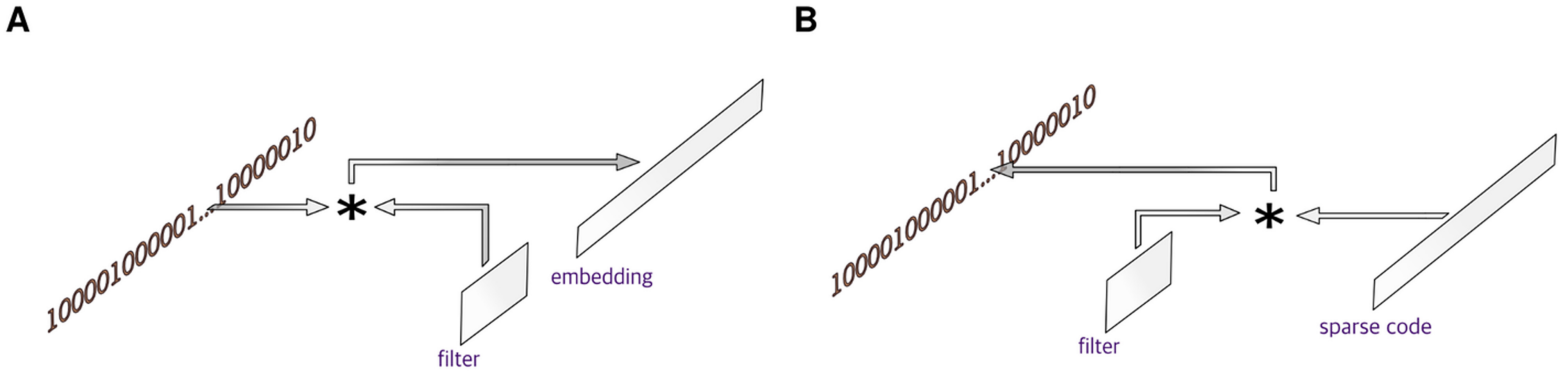
(A) Convolutional neural network (CNN). (B) Sparse representation. The symbol * denotes the convolution. In CNN, the filters are convolved with the one-hot encoded DNA string to generate an embedding for downstream purposes, e.g. predicting the bigWig coverage. In sparse representations, the filters are convolved with the sparse code, where the sparse code plays the role of an indicator, indicating where the filters should represent the DNA string.

More recently, motifs are inferred from deep learning methodologies, epitomized by the use of convolutional neural network (CNN). Compared with traditional methods, inferring the motifs is less straightforward: some approaches identify the motifs as the filters in the first convolutional layers, while others use model agnostic explanation methods such as DeepLift or SHapley Additive exPlanations (SHAP) (Lundberg and Lee 2017, Shrikumar *et al.* 2017). Furthermore, the filters in CNNs are convolved with DNA strings to create embeddings (Fig. 1), which by construction are primarily intended for building regressors, e.g. predicting the bigWig coverage (Kelley *et al.* 2016, Avsec *et al.* 2021a,b, Yuan and Kelley 2022). Therefore, identifying all conserved patterns in datasets is typically not the main objective of CNNs. Consequently, the motifs predicted by CNNs are not significantly different from those predicted by traditional methods, and a principled, unifying model that effectively captures the long, gapped, and flexible motifs present in the dataset is still lacking.

In this work, we introduce a hierarchical sparse representation as a principled framework for motif discovery. Our method is capable of capturing statistically significant long, gapped, and flexible motifs in addition to the primary motifs in the dataset. These motifs are often longer than 30 base pairs in length, beyond the computable range for methods based on k-mer enumerations such as STREME and HOMER. In addition, as sparse representations are interpretable by design, we alleviate the need for explanation methods like SHAP. Our method can efficiently scale to large datasets containing hundreds of thousands of DNA strings, requiring only a moderate amount of computational resources.

## 2 Materials and methods

### 2.1 Notation

We refer to DNA strings as strings defined on Σ={A,C,G,T}. The *i*-th element of a vector v is denoted by v[i]. Vectors and matrices are denoted by boldface letters, while scalars are non-bold. We use the notation x≥0 to indicate that all components of the vector or matrix x are non-negative. The function |·| returns the cardinality of a finite set. The norms $||\cdot |{|}_{F},\;||\cdot |{|}_{2},\;||\cdot |{|}_{1}$, and $||\cdot |{|}_{0}$ denote the Frobenius norm, $\ell_{2}$ norm, $\ell_{1}$ norm, and $\ell_{0}$ norm, respectively.

### 2.2 Problem formulation

**Figure 2.**
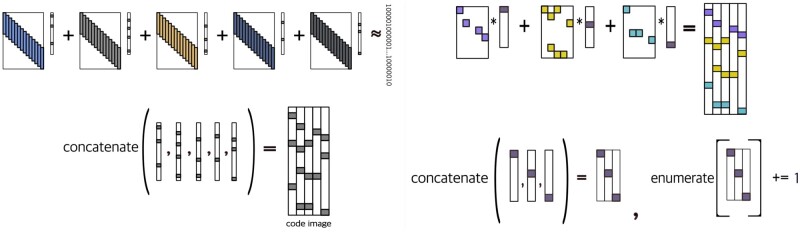
We show how we enumerate at the image level, with a single one-hot encoded DNA string as the input: (A) we first obtain a sparse representation of the one-hot encoded DNA string as a sum of linear convolutions. Next, we concatenate the collection of the sparse code in this sparse representation as an image. We refer to this image as a *code image*. (B) We obtain a sparse representation of the code image. Using the sparse representation of the code image, we enumerate the configurations within the sparse code.

Our method begins with a simple idea: we induce a sparse representation of a DNA string by assuming that each one-hot encoded DNA string $s_{n}$ can be represented as a finite sum of linear convolutions, i.e. where $d_{m}$ represents the features, often called filters, and $x_{mn}$ is the corresponding sparse vector of $s_{n}$, known as the sparse code (Bristow *et al.* 2013, Heide *et al.* 2015, Wohlberg 2016, Dumitrescu and Irofti 2018, Garcia-Cardona and Wohlberg 2018). We consider the filters can be reshaped as ℓ-column position frequency matrices (PFMs) that capture the frequency of nucleotides at each position of a length-ℓ DNA string, i.e. which resolves the scaling ambiguity between the filters and the sparse code. A key insight we present is that the sparse code can be likened to images. We refer to such images as *code images*, and we generate one for each string $s_{n}$ by horizontally concatenating the sparse code $x_{mn}$ for all *m* (Fig. 2A). Using the set of DNA strings and their corresponding code images, we proceed to construct a sparse representation specifically tailored to represent these images (Fig. 2B). By doing so, we are able to identify a more extensive and diverse set of patterns in the DNA strings by enumerating the combinations within the sparse code. This technique, which we refer to as *enumerating at the image level*, yields a broader range of significant patterns than by enumerating the k-mers in the DNA strings.


(1) $$s_{n}\approx \sum_{m}d_{m}*x_{mn}$$

$$P_{\ell }=\left\{p\in R_{+}^{4\times \ell }:\sum_{\alpha \in \Sigma }p[\alpha ,\kappa ]=1,\kappa =1,\ldots ,\ell \right\},$$

To start the motif discovery of DNA strings $s_{1},\ldots ,s_{N}$, we first tackle the following problem as a precursor to enumerating at the image level:


(2) $$\begin{array}{c} \underset{\left\{\overset{x_{mn},y_{mn},z_{kn}}{F_{k},\;\;d_{m}}\right\}}{\text{argmin}}\frac{1}{2}\sum_{n}\|\sum_{m}d_{m}*x_{mn}+{\tilde{d}}_{m}*y_{mn}-s_{n}{\|}_{2}^{2}\\ \begin{array}{c} +\;\mu \sum_{k}{\left\|F_{k}\right\|}_{1}+\lambda \left(\sum_{n,m}{\left\|x_{mn}\right\|}_{1}+{\left\|y_{mn}\right\|}_{1}\right)\\ \text{subject to}\;\;\sum_{k}F_{k}*z_{kn}=T\left(X_{\cdot n},Y_{\cdot n}\right)\\ F_{k}\geq 0,{\left\|F_{k}\right\|}_{F}=1\;\forall k=1,\ldots ,K\\ Z_{\cdot n}\in \left\{Z_{\cdot n}:||Z_{\cdot n}|{|}_{\;0}\leq \alpha \right\}\;\forall n=1,\ldots ,N\\ \mathrm{reshape}(d_{m})\in P_{\ell }\;\forall m=1,\ldots ,M\\ x_{mn},y_{mn}\geq 0\;\forall m=1,\ldots ,M,n=1,\ldots ,N \end{array} \end{array}$$

In problem 2, our first goal is to approximate DNA strings by a sum of convolutions while promoting the sparsity in the code vectors $x_{mn},\;y_{mn}$. The filter $d_{m}$ is reversed to obtain ${\tilde{d}}_{m}$, enabling the consideration of patterns in both the forward and complementary strands of DNA strings. Our second goal in problem 2 is to extract patterns at the image level (Fig. 2B). To be precise, the matrices $X_{\cdot n},\;Y_{\cdot n}$, and $Z_{\cdot n}$ are constructed by horizontally concatenating the corresponding code vectors $x_{1n},\ldots ,x_{Mn},\;y_{1n},\ldots ,y_{Mn}$, and $z_{1n},\ldots ,z_{Kn}$, respectively. Similarly, the matrix $\left(X_{\cdot n},,Y_{\cdot n}\right)$ is formed by concatenating $X_{\cdot n}$ and $Y_{\cdot n}$ horizontally. The transformed version of $\left(X_{\cdot n},Y_{\cdot n}\right)$ through a linear transform T is denoted as $T\left(X_{\cdot n},Y_{\cdot n}\right)$. To ensure a parsimonious representation of the transformed image $T\left(X_{\cdot n},Y_{\cdot n}\right)$, we constrain the matrix $Z_{\cdot n}$ to be at most *α*-sparse and require the sum of convolutions $\sum_{k}F_{k}*z_{kn}$ to match $T\left(X_{\cdot n},Y_{\cdot n}\right)$. Last, we impose a sparsity penalty on the filters $F_{k}$ so that they capture the most salient features in the images $T\left(X_{\cdot n},Y_{\cdot n}\right)$.

### 2.3 Obtaining the sparse representation: the classical way

We can design iterative algorithms to solve problem 2 by alternatively minimizing the sparse code $\left\{x_{mn},y_{mn},z_{kn}\right\}$ and the filters $\left\{F_{k},d_{m}\right\}$. To do so, we define $L_{\cdot n}$ and $R_{\cdot n}$ as the left and right halves of the image $\sum_{k}F_{k}*z_{kn}$, respectively, and for simplicity, let T be the identity transform. With the filters $\left\{F_{k},d_{m}\right\}$ held fixed, we apply Alternating Direction Method of Multipliers (ADMM) (Boyd 2010) to problem 2 to solve the sparse code $\left\{x_{mn},y_{mn},z_{kn}\right\}$: where $L_{mn},R_{mn}$ are the *m*-th column of $L_{\cdot n}$ and $R_{\cdot n}$, respectively. The scaled dual variables are $\Gamma_{\cdot n}$ and ${ϒ}_{\cdot n}$, and the penalty parameter is *ρ*. The function $1_{\alpha }(\cdot )$ is an indicator function that projects the input matrix into the space of matrices with at most *α* non-zero elements.


(3) $$\begin{array}{c} x_{mn}^{\;t+1},y_{mn}^{\;t+1}=\\ \underset{\left\{x_{mn}\geq \;0,\;y_{mn}\geq \;0\right\}}{\text{argmin}}\frac{1}{2}{\left\|\sum_{h}d_{h}*x_{mn}+{\tilde{d}}_{h}*y_{mn}-s_{n}\right\|}_{2}^{2}\\ \;\;\;\;\;+\lambda \left({\left\|x_{mn}\right\|}_{1}+{\left\|y_{mn}\right\|}_{1}\right)\\ \;\;\;+\frac{\rho }{2}({\left\|L_{mn}^{\;t}-x_{mn}+\Gamma_{mn}^{\;t}\right\|}_{2}^{2}\\ \;\;\;+{\left\|R_{mn}^{\;t}-y_{mn}+{ϒ}_{mn}^{\;t}\right\|}_{2}^{2}),\\ \;\;\forall m=1,\ldots ,M,n=1,\ldots ,N \end{array}$$

(4) $$\begin{array}{c} Z_{\cdot n}^{\;t+1}=\underset{\left\{Z_{\cdot n}\right\}}{\text{argmin}}1_{\alpha }\left(Z_{\cdot n}\right)\\ +\frac{\rho }{2}{\left\|\sum_{k}F_{k}*z_{kn}-\left(X_{\cdot n}^{\;t+1},Y_{\cdot n}^{\;t+1}\right)+\left(\Gamma_{\cdot n}^{\;t},{ϒ}_{\cdot n}^{\;t}\right)\right\|}_{F}^{2},\\ \forall n=1,\ldots ,N \end{array}$$

(5) $$\begin{array}{c} \left(\Gamma_{\cdot n}^{\;t+1},{ϒ}_{\cdot n}^{\;t+1}\right)=\left(\Gamma_{\cdot n}^{\;t},{ϒ}_{\cdot n}^{\;t}\right)+\sum_{k}F_{k}*z_{kn}^{\;t+1}-\left(X_{\cdot n}^{\;t+1},Y_{\cdot n}^{\;t+1}\right),\\ \forall n=1,\ldots ,N \end{array}$$

The solution to equation (3) can be obtained by alternatively solving $x_{mn}$ and $y_{mn}$ using iterative shrinkage thresholding algorithm (ISTA), i.e. for all *m*, *n*, where ⊛ is the cross correlation, $S_{\lambda \eta^{t}}^{+}(\cdot )$ is the non-negative soft-threshold operator, and $\eta^{\;t}$ the step size at *t*. We consider projected gradient descent to solve equation 4. Here, an update step for all *n* is where ${\text{Project}}_{\alpha }(\cdot )$ keeps at most *α* largest magnitude components and zeros out the rest, $G_{Z}$ the gradient of the penalty term of equation (4), and $\gamma^{\;t}$ is the step size at *t*. On the other hand, by applying block coordinate descent with method of multipliers to problem 2 with the sparse code $\left\{x_{mn},y_{mn},z_{kn}\right\}$ held fixed, we obtain the following iterative algorithm to solve the filters $\left\{F_{k},d_{m}\right\}$: where *τ* is the penalty term and $\Theta_{\cdot n}$ are the scaled dual variables. We can solve equation (8) via mirror descent (Beck and Teboulle 2003). Because we can reshape each $d_{m}$ into a ℓ-column PFM by assumption, a way to express each component of $d_{m}$ is $d_{m}[4(j_{1}-1)+j_{2}]$ for $j_{1}=1,\ldots ,\ell ,j_{2}=1,\ldots ,4$. We define the distance function *ψ* associated with the mirror descent as the sum of the negative entropy of each column of the reshaped filter $d_{m}$. This function is expressed as:


(6) $$\begin{array}{c} x_{mn}^{\;t+1}=S_{\lambda \eta_{t}}^{+}(x_{mn}^{\;t}-\eta^{\;t}d_{m}\;⊛\;△-\rho \left(x_{mn}^{\;t}-L_{mn}^{\;t}-\Gamma_{mn}^{\;t}\right))\\ y_{mn}^{\;t+1}=S_{\lambda \eta_{t}}^{+}(y_{mn}^{\;t}-\eta^{\;t}{\tilde{d}}_{m}\;⊛\;△-\rho \left(y_{mn}^{\;t}-R_{mn}^{\;t}-{ϒ}_{mn}^{\;t}\right))\\ \text{with}\;△=\sum_{h}d_{h}*x_{hn}^{\;t}+{\tilde{d}}_{h}*y_{hn}^{\;t}-s_{n} \end{array}$$

(7) $$Z_{\cdot n}^{\;t+1}={\text{Project}}_{\alpha }\left(Z_{\cdot n}^{\;t}-\gamma^{\;t}G_{Z}\right)$$

(8) $$\begin{array}{c} d_{m}^{\;t+1}=\underset{\text{reshape}(d_{m})\;\in \;P_{\ell }}{\text{argmin}}\frac{1}{2}\sum_{n}{\left\|\sum_{m}d_{m}*x_{mn}+{\tilde{d}}_{m}*y_{mn}-s_{n}\right\|}_{2}^{2},\\ \forall m=1,\ldots ,M \end{array}$$

(9) $$\begin{array}{c} F_{k}^{\;t+1}=\underset{F_{k}\;\geq \;0}{\text{argmin}}\mu \sum_{k}{\left\|F_{k}\right\|}_{1}\\ +\frac{\tau }{2}\sum_{n}{\left\|\sum_{k}F_{k}*z_{kn}-\left(X_{\cdot n},Y_{\cdot n}\right)+\Theta_{\cdot n}^{\;t}\right\|}_{F}^{2},\\ \forall k=1,\ldots ,K \end{array}$$

(10) $$\begin{array}{c} \Theta_{\cdot n}^{\;t+1}=\Theta_{\cdot n}^{\;t}+\sum_{k}F_{k}*z_{kn}-\left(X_{\cdot n},Y_{\cdot n}\right),\\ \forall n=1,\ldots ,N \end{array}$$

$$\begin{array}{c} \psi (d_{1},\ldots ,d_{M})=\\ \sum_{m=1}^{M}\sum_{j_{1}=1}^{\ell }\sum_{j_{2}=1}^{4}d_{m}[4(j_{1}-1)+j_{2}]\;\text{log}\;(d_{m}[4(j_{1}-1)+j_{2}]). \end{array}$$

Therefore, to update each filter $d_{m}$ with all components indexed by *j*_1_ and *j*_2_ in the mirror descent, we have the following expression: with $\pi^{\;t}$ the step size at *t*, and $g_{m}^{\;t}$ the gradient of $d_{m}$ in equation (8). We solve equation (9) by ISTA for each filter $F_{k}$: and then normalize so that each ${\left\|F_{k}\right\|}_{F}=1$. Here, $\omega^{\;t}$ is the step size at *t* and $S_{\mu \omega^{t}}^{+}(\cdot )$ is the non-negative soft-threshold operator.


(11) $$\begin{array}{c} d_{m}^{\;t+1}[4(j_{1}-1)+j_{2}]=\\ \frac{d_{m}^{\;t}[4(j_{1}-1)+j_{2}]\;\text{exp}(-\pi^{\;t}g_{m}^{\;t}[4(j_{1}-1)+j_{2}])}{\sum_{j_{3}=1}^{4}d_{m}^{\;t}[4(j_{1}-1)+j_{3}]\;\text{exp}(-\pi^{\;t}g_{m}^{\;t}[4(j_{1}-1)+j_{3}])} \end{array}$$

(12) $$\begin{array}{c} F_{k}^{\;t+1}=S_{\mu \omega^{t}}^{+}\left(F_{k}^{\;t}-\omega^{\;t}\tau \sum_{n}z_{kn}⊛△\prime \right)\\ \text{with}\;△\prime =\sum_{k\prime }F_{k\prime }^{\;t}*z_{k\prime n}-\left(X_{\cdot n},Y_{\cdot n}\right)+\Theta_{\cdot n}^{\;t} \end{array}$$

### 2.4 Obtaining the sparse representation by deep unfolding

Rather than relying on the algorithm from Section 2.3 to obtain the sparse representation, we adopt a different approach called *deep unfolding* (Gregor and LeCun 2010, Monga *et al.* 2021). The deep unfolding approach employs a neural network that parameterizes the iterates of an iterative algorithm as its forward pass, which obtains an approximate representation of the problem and has been shown to achieve significantly faster convergence in practice (Gregor and LeCun 2010).

We use deep unfolding to obtain an approximate sparse representation in problem 2. To construct our network, we use the iterates from Section 2.3, with the sparse code $\left\{x_{mn},y_{mn},z_{kn}\right\}$ and the scaled dual variables $\left\{\Gamma_{\cdot n},{ϒ}_{\cdot n},\Theta_{\cdot n}\right\}$ in the network initialized to be zero. Next, the equations (6), (7), (11), and (12) can be implemented as non-linearities of the layers in the network. We construct the first $2\times K_{1}$ layers by interleaving the iterates of equations (6) and (7), and the subsequent $2\times K_{2}$ layers by executing the iterates of equations (11) and (12). The parameters of the our network are the filters $\left\{d_{m},F_{k}\right\}$, the sparsity inducing parameters {λ,μ}, the penalty parameters {ρ,τ}, and the stepsizes $\left\{\eta^{\;t_{1}},\gamma^{\;t_{1}},\pi^{\;t_{2}},\omega^{\;t_{2}}:t_{1}=1,\ldots ,K_{1},t_{2}=1,\ldots ,K_{2}\right\}$, learned by training the network with backpropagation.

**Figure 3.**
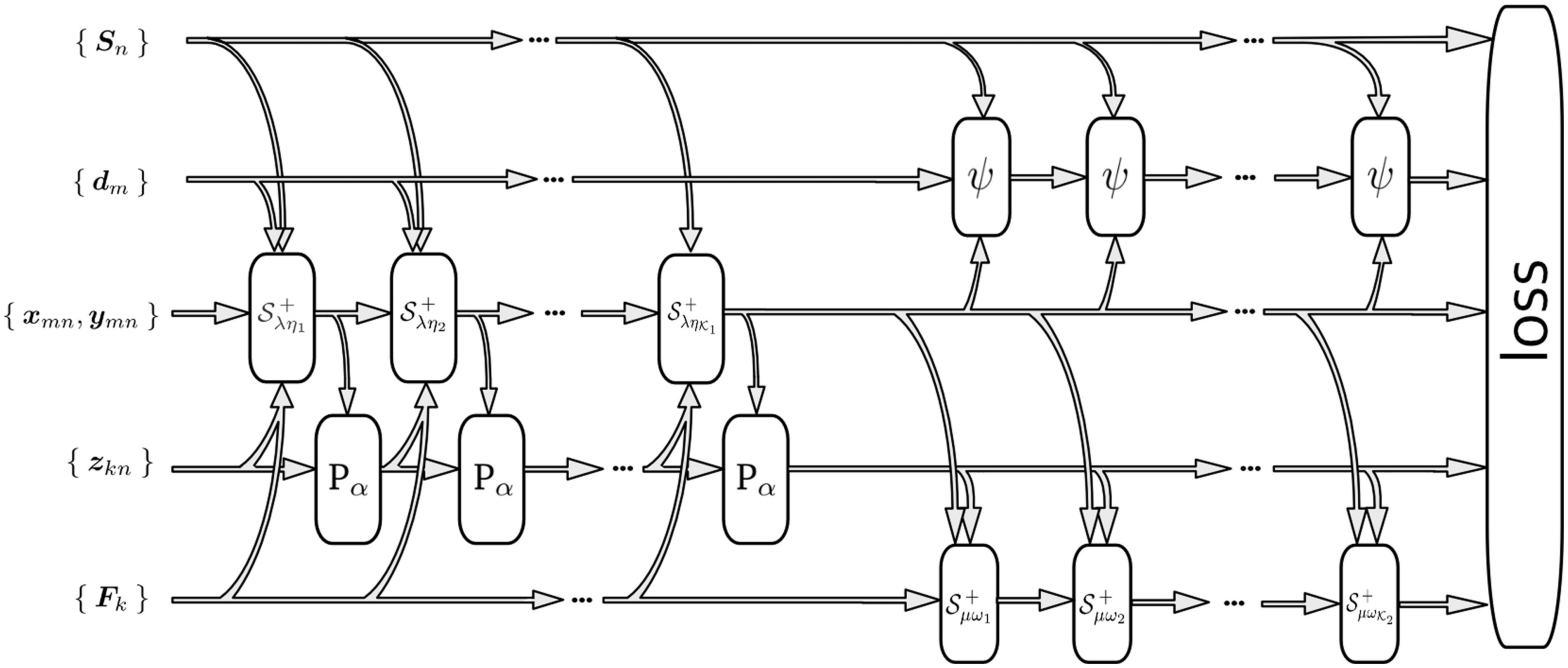
The unfolded network obtained by parameterizing the iterates derived in Section 2.3.

Once the network completes its forward pass, we define the loss of the network as where the sparse code $\left\{x_{mn}^{\;K_{1}},y_{mn}^{\;K_{1}},z_{kn}^{\;K_{1}}\right\}$ are from the final output of the first $2\times K_{1}$ layers, and the filters $\left\{d_{m}^{\;K_{2}},F_{k}^{\;K_{2}}\right\}$ are from the final output of the subsequent $2\times K_{2}$ layers of the network. An illustration of the unfolded network is shown in Fig. 3.


$$\begin{array}{c} \frac{1}{N}\sum_{n}[{\left\|\sum_{m}d_{m}^{\;K_{2}}*x_{mn}^{\;K_{1}}+{\tilde{d}}_{m}^{\;K_{2}}*y_{mn}^{\;K_{1}}-s_{n}\right\|}_{2}^{2}\\ +{\left\|\sum_{k}F_{k}^{\;K_{2}}*z_{kn}^{\;K_{1}}-T\left(X_{\cdot n}^{\;K_{1}},Y_{\cdot n}^{\;K_{1}}\right)\right\|}_{F}^{2}] \end{array}$$

### 2.5 Enumerating at the image level

After the network is trained, we can retrieve the sparse code of the code images, i.e. the set $\left\{Z_{\cdot n}\right\}$ by a network forward pass. Similar to the seeding phase in methods such as STREME and HOMER, our motif discovery also performs enumerations. Yet, instead of enumerating the oligonucleotides, we enumerate the combinations of the sparse code components in each $Z_{\cdot n}$, for n=1,…,N. Specifically, we count how $q\in Z_{+}$ components are arranged in each $Z_{\cdot n}$. Given that $Z_{\cdot n}$ has at most *α* components, the number of operations required to enumerate all possible combinations of *q* components in each $Z_{\cdot n}$ is at most $\left(\begin{array}{c} \alpha \\ q \end{array}\right)$. This is a relatively small number, especially considering that we restrict both *α* and *q* to be small integers.

We refer to each combination of *q* components and its spatial information a *configuration*. The configuration is defined as a tuple: where the numbers $f_{\left(1\right)},f_{\left(2\right)},\ldots ,f_{\left(q\right)}$ are the *q* filter-indices of filters $\left\{F_{k}\right\}$ directly inferred from $Z_{\cdot n}$ in the combination, sorted by their spatial occurrences in the DNA string $s_{n}$. The numbers $d_{\left(1\right)},d_{\left(2\right)},\ldots ,d_{\left(q-1\right)}$ are the distances (number of nucleotides) in between their neighboring components in the configuration. We store the configurations as keys in a hash table H. The value associated with each key in H is a collection of DNA strings of the same length, each obtained by taking the DNA string in the region covered by the particular configuration, as we perform the enumerations through DNA strings $s_{1},\ldots ,s_{n}$. Thus, each set of the DNA strings associated with a key defines a multiple sequence alignment (MSA), and corresponds to a position weight matrix (PWM).


$$\left(f_{\left(1\right)},d_{\left(1\right)},f_{\left(2\right)},d_{\left(2\right)},\ldots ,d_{\left(q-1\right)},f_{\left(q\right)}\right)$$

We select up to *J* most enriched PWMs in the hash table H as the motifs. As multiple keys in H may correspond to similar motifs, we reduce such redundancy by merging the similar PWMs using the average log-likelihood ratio (Wang and Stormo 2003). We report the resulting PWMs $P_{1},\ldots ,P_{J\prime }$ as the discovered motif in the dataset.

### 2.6 Soft versus hard clustering representation of the motifs

Since a PWM represents an average and proteins can have distinct binding preferences, the motif discovery problem is, in a sense, similar to the clustering problem. Soft-clustering scenarios, such as mixtures of Gaussians, allow a point to belong to multiple clusters, exhibiting characteristics that coexist in each of them. This situation frequently arises in motif discovery, where, for instance, the primary motif may frequently occur by itself but occasionally appears twice in certain DNA strings in the dataset.

In this work, we present our result in the soft-clustering representation. Unlike most traditional motif discovery methods that use hard clustering representations, which use PWMs composed of mutually exclusive DNA substrings, our method employs soft clustering representations that allow PWMs derived by multiple sequence alignment to share DNA substrings with other PWMs. We provide a detailed comparison of both approaches and their trade-offs in Supplementary File S1 Section C.

### 2.7 Implementation

#### 2.7.1 Hyperparameters

We implement our model with $M=|\left\{d_{m}\right\}|=50$ filters for the sparse representation of DNA strings $\left\{s_{N}\right\}$, where each filter (a PFM) $d_{m}$ covers ℓ=8 nucleotides. Additionally, we use $K=|\left\{F_{k}\right\}|=24$ filters for code images, where each filter $F_{k}$ can cover 12×(2×M) pixels. To limit the number of nonzero elements in each code image $Z_{\cdot n}$, we set *α* to 32. We set the operator T as a scaling operator, i.e. T(M)=βM, where *β* = 100 to ensure numerical stability. Our unfolded network is trained using AdaBelief (Zhuang *et al.* 2020), with a batch size of 6 DNA strings. We set $K_{1}$ to 6 interleaved iterations on the sparse code and $K_{2}$ to 3 iterations on the filters. This parameterization results in a network, i.e. Fig. 3, consisting of 30, 421 parameters, which is much smaller in size compared with current mainstream deep learning models. We set *q *=* *3 for the configurations, which results in at most $\left(\begin{array}{c} \alpha \\ q \end{array}\right)=4,960$ counting operations for each retrieved code image $Z_{\cdot n}$. We select up to *J *=* *1000 most enriched motifs from the stored enumerations in the hash table H to display in the results.

#### 2.7.2 Motif significance

We randomly select DNA strings and set aside 15% of them as a test set that is not used during training. For each motif discovered and indexed by *j*, we follow this procedure: Let *N_T_* denote the total number of available positions in the test set, where *N_t_* is the number of DNA strings in the test set, and *L* is the length of each string. We define $N_{T}=N_{t}L$. The number of hits of the *j*-th motif in the test set is denoted by *τ_h_*, and the number of misses is denoted by $\tau_{m}=N_{T}-\tau_{h}$. A hit at a position is defined as the positions occupied by the PWM that scored above its threshold. The score threshold for each PWM is determined using the approximation algorithm pvalue2score with a *P*-value of 1e−3 (Touzet and Varré 2007). Hits and misses for the control set are similarly defined as *c_h_* and *c_m_*, respectively. The control set is constructed by shuffling the DNA strings in the test set such that the 2-mer frequency is preserved. We then perform Fisher’s exact test to test the null hypothesis that the odds ratio $\left(\tau_{h}/\tau_{m})/(c_{h}/c_{m}\right)$ is one, against the alternative hypothesis that they are not equal.

#### 2.7.3 Motif occurrences

We define the number of instances of a motif as the number of position that a PWM scores above its score threshold, i.e. the number of hits (Section 2.7.2) in the dataset.

## 3 Results

**Figure 4.**
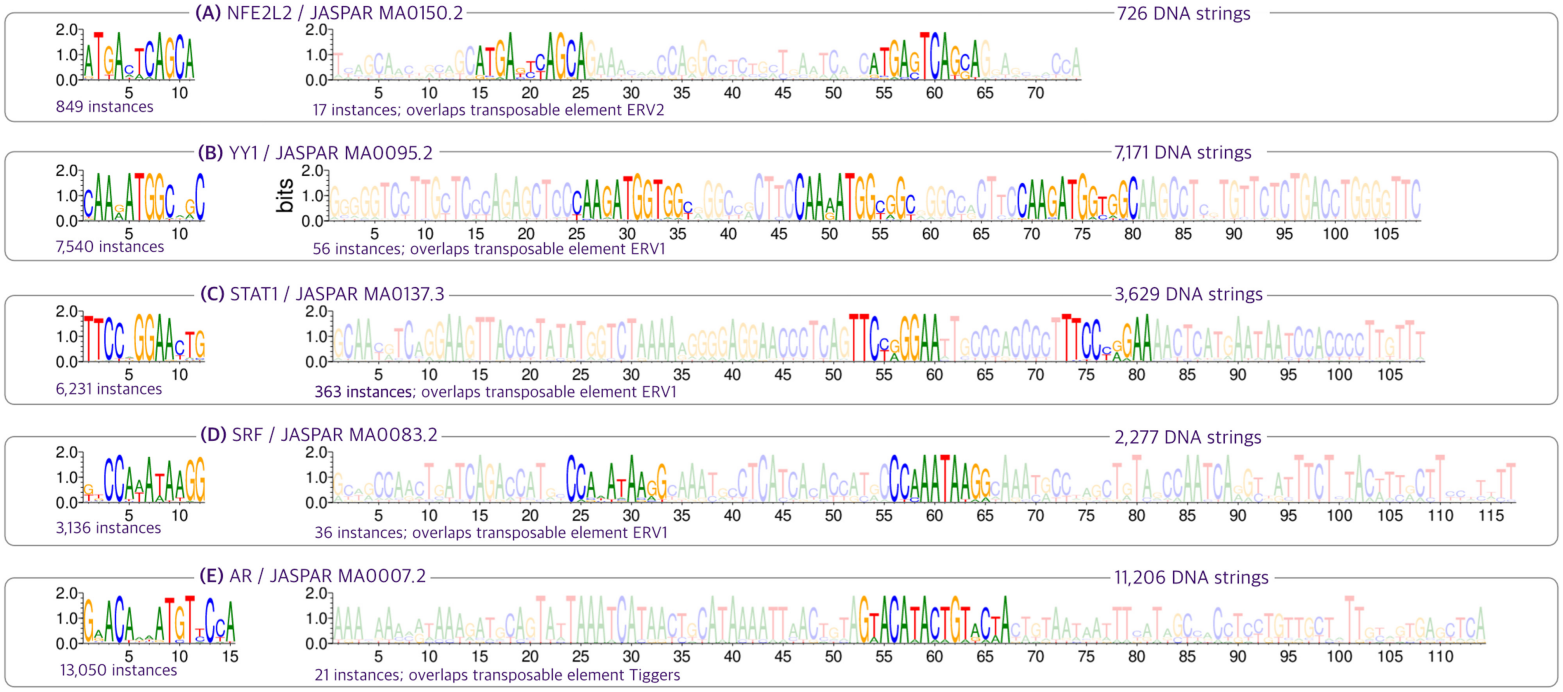
For each dataset, we list the TF/JASPAR-ID and the number of DNA strings in the dataset. We show how the primary motifs are embedded in the repetitive elements, with the primary motifs on the left and their overlap on the right. Below each sequence logo, we show the number of instances that occurred throughout the dataset. In short, (A) NFE2L2 overlaps the ERV2. (B, C, and D) YY1, STAT1, and SRF overlaps the ERV1. (E) AR overlaps Tiggers. Each transposable element is validated by taking the consensus string of each PWM and then search to confirm via Dfam (Hubley *et al.* 2016). For more, see Supplementary File S1 Section E.

**Figure 5.**
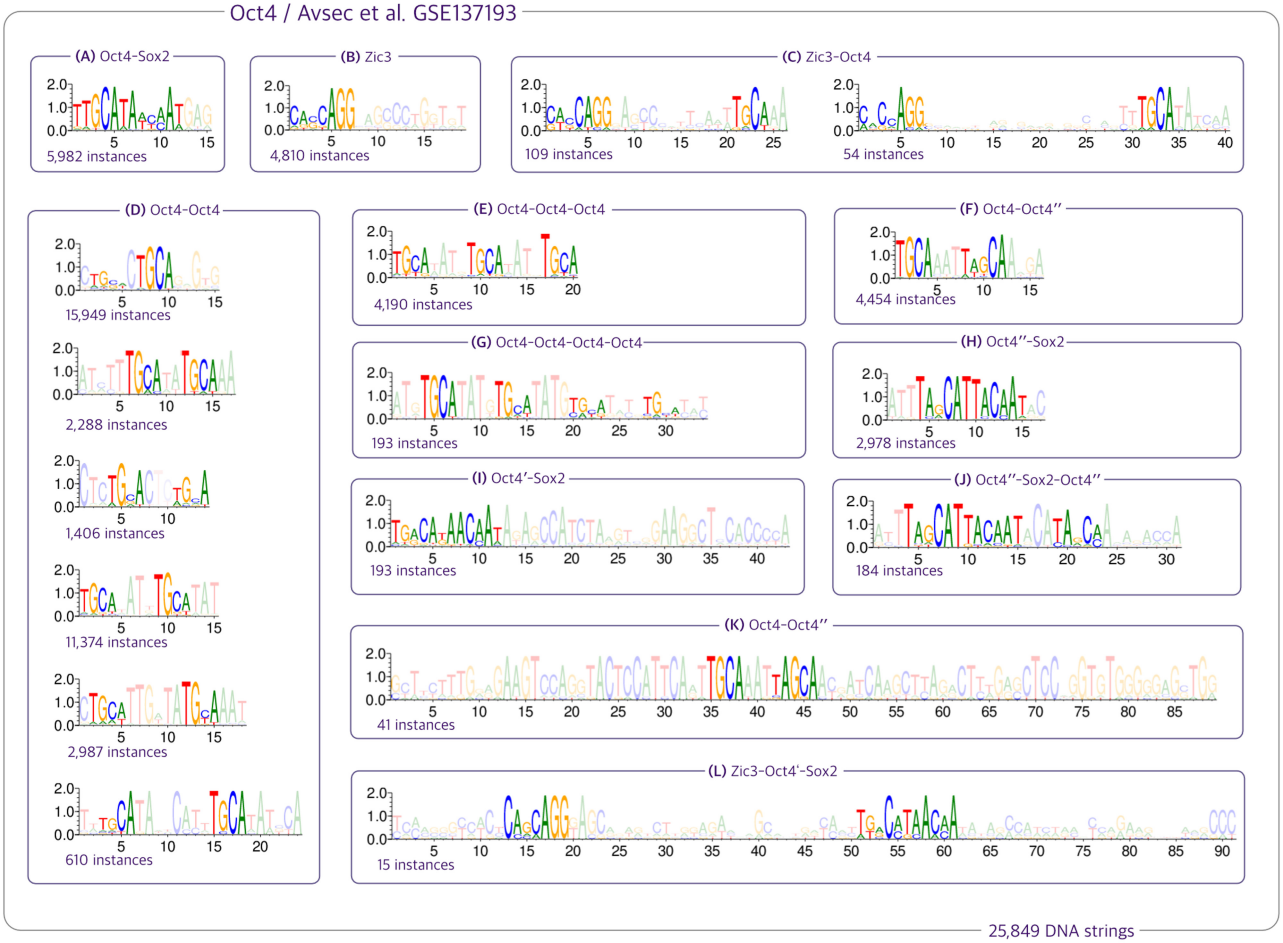
(A, B, and D) We validated the presence of motifs reported in the ChIP-Nexus experiment (GEO: GSE137193) conducted by (Avsec *et al.* 2021a), such as Oct4-Sox2, Oct4-Oct4, and Zic3. (D) Our analysis revealed new insights into the Oct4-Oct4 motif, including the potential for variable spacing between the half-sites TGCA, ranging from 1 to 8 nucleotides. (E, G, C) We also observed that Oct4 can co-occur up to 4 times with equal-length spacers of TATG, and it is frequently associated with Zic3 factors. (F, H, I, J, K, L) Furthermore, we identified two minor variants of Oct4, which we designate as Oct4′ and Oct4″, due to the insertion of a nucleotide A either upstream or downstream of the central nucleotide G in Oct4. Our additional findings for Sox2, Klf4, and Nanog pluripotency factors from (Avsec *et al.* 2021a) are presented in Supplementary File S1 Section F.

**Figure 6.**
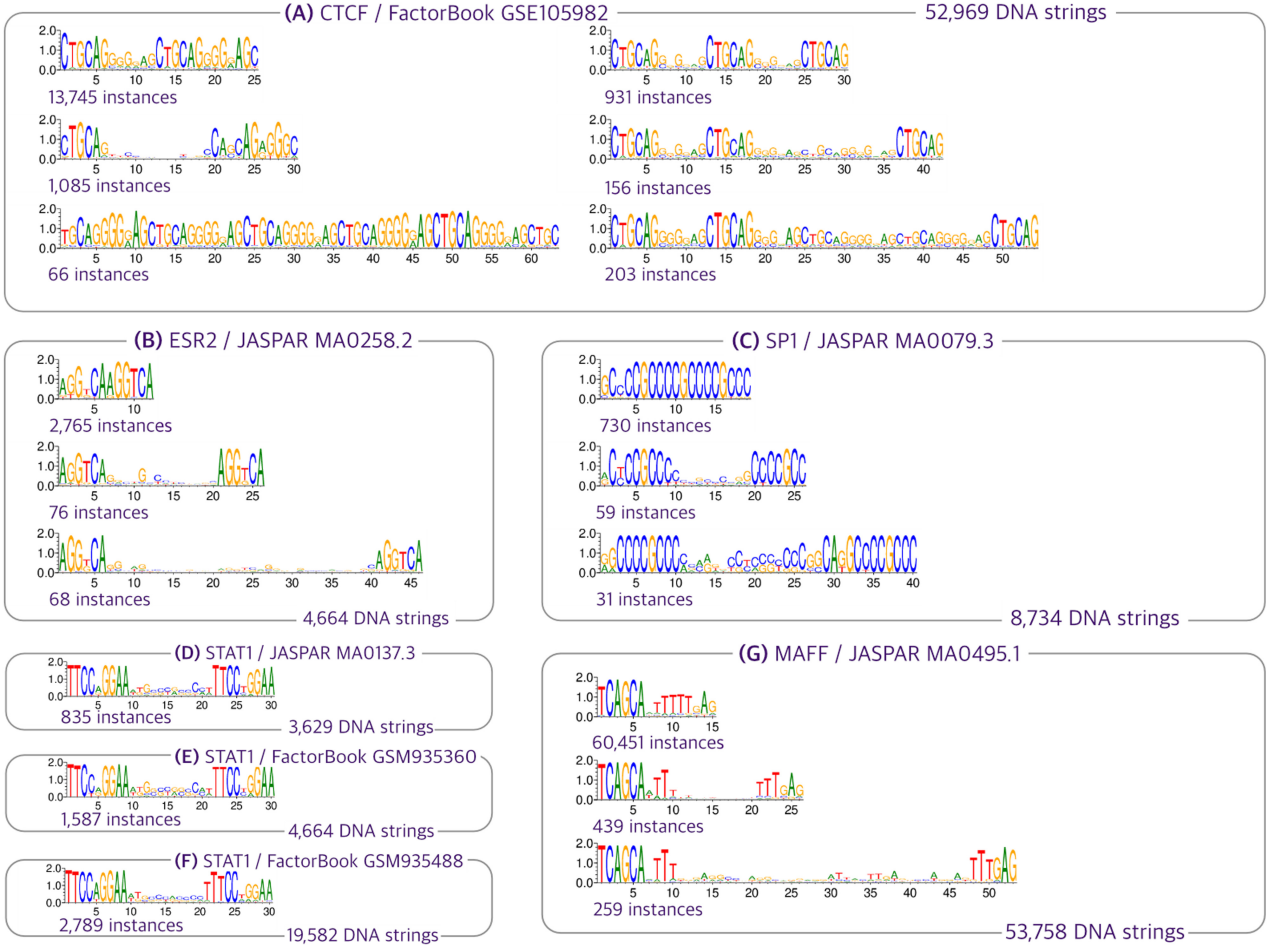
We present a selection of gapped motifs, chosen from the many we discovered in each dataset (labeled with TF/GEO accession number or TF/JASPAR-ID). (A) Notably, we discovered the presence of the upstream motif CTGCAG of CTCF (Zuo *et al.* 2023); on the right, we note that the upstream motif of the core motif occur independently of the primary motif of CTCF. (C) Our analysis reveals that the primary motif of SP1 and ESR2 exhibits repetition with spacers of varying lengths. We note that ESR2 variable spacing correspond to the findings reported in (Siggers and Gordân, 2014). (D, E, F) In the case of STAT1, we observe a fixed spacing across all three datasets. (G) We present a gapped motif discovered in MAFF, which demonstrates our method’s capability to capture gapped motifs with spacers longer than 40 bp. To avoid clutter, we display only motifs with the smallest, middle, and largest spacers, as determined by their pairwise distance (number of nucleotides) for ESR2, SP1, and MAFF. A full result of our motif discovery is in Supplementary File S1 Section F.

We take experimental datasets from JASPAR, FactorBook, ReMap, and Avsec *et al.* (Weirauch *et al.* 2014, Avsec *et al.* 2021b, Castro-Mondragon *et al.* 2022, Hammal *et al.* 2022, Pratt *et al.* 2022) to conduct motif analysis, for which we detailed our data processing steps in Supplementary File S1 Section A. In our analysis, we characterize all the motifs as position weight matrices (PWMs). All motifs presented in this section (motifs shown in Figs 4–6) are statistically significant with a *P*-value <1e−6, as determined by the Fisher exact test (Section 2.7.2). Additionally, we make the following characterizations:

- *Primary motif*: the short (often 6–12bp), ungapped, PWM that correspond to the core binding sites of a transcription factor (TF).
- *Long motif*: the long (often longer than 30bp) PWM with uniformly high information content columns, e.g. Fig. 4; these motifs, like gapped motifs, can also characterize multiple binding sites of the same or different TFs.
- *Gapped motif*: the PWM that contain groups of consecutive high information content columns separated by low information content columns, e.g. PWMs in Fig. 6.

Our analysis of the datasets reveals a large presence of long motifs and gapped motifs in these experimental datasets. In particular, among the 91 ChIP-Seq datasets we selected from JASPAR, 50 of them contain long motifs that are transposable elements, as we verified in the Dfam database (Hubley *et al.* 2016) (Supplementary File S1 Section E). Moreover, gapped motifs were identified in many datasets from diverse sources, including 89 out of 198 datasets in JASPAR across various experiment types including ChIP-seq, DAP-seq, SELEX, PBM, and ChIP-Chip. This large presence of long motifs and gapped motifs in our analysis is noteworthy, as they are often overlooked in motif discovery and may have important implications in transcriptional regulation. In the following sections, we will highlight the discovery of gapped and long motifs. We skip the results on primary motif discovery as our method almost always find them (Supplementary File S1 Section F), and it is straight-forward problem tackled by many well-established methods.

### 3.1 Long motifs

A central theme that frequently occurs in long motif discovery is the overlap between the long motifs and the primary motifs. These overlappings present a significant challenge for traditional motif discovery methods, discussed in Section 4.1. In qualitative terms, the primary motifs can either be “embedded” within the long motifs or “compounded” in proximity to other motifs in the dataset.

#### 3.1.1 “Embedded” motifs

Our method simultaneously discovers the primary motifs and the long motifs that embed these primary motifs, as shown in Fig. 4. We observe many such cases, especially in ChIP-Seq datasets, where TF binding sites in vivo can often overlap with repetitive elements. Our result reveals a relationship between primary motifs and repetitive elements, and suggests that the use of *repeat masking* is not strictly necessary for motif discovery.

#### 3.1.2 “Compound” motifs

Our analysis shows that compound motifs are frequently seen in ChIP-Seq datasets. These findings suggest that our method effectively identifies binding sites including those that work in conjunction with the TF of interest, as TFs often work in concert with other TFs to regulate gene expression in vivo. We show in Fig. 5 several compound motifs that we identified in a ChIP-Nexus experiment exploring the localization of Oct4 pluripotency factor, using experimental data from (Avsec *et al.* 2021b).

### 3.2 Gapped motifs

Our method detects several gapped motifs that have been previously reported in the literature, shown in Fig. 6. For instance Zuo *et al.* (2023) report that traditional motif discovery methods have been shown to underestimate the binding sites of CTCF, a zinc finger protein containing 11 zinc finger domains, resulting in a paradox known as *long fingers but short motifs*. This paradox highlights that the full binding sites of evolutionarily conserved zinc finger domains may follow an irregular structure that is not easily detected by traditional methods. Our method successfully identifies the upstream motif TGCAG of the core binding sites of CTCF, as reported in (Zuo *et al.* 2023), shown in Fig. 6A. In addition, we confirm that ESR2, a Nuclear Receptor factors binds with its half sites AGGTCA (Khorasanizadeh and Rastinejad 2001, Siggers and Gordân 2014), which we found to be separated by a spacer up to 36 nucleotides, shown in Fig. 6B.

Notably, our method exhibits high sensitivity in quantifying the spacers within gapped motifs, providing detailed insights into gapped motifs’ composition. For example, we identified a gapped motif in the MAFF factor from JASPAR that exhibits a gap between the primary motif and its partial complement, with a total of 26 spacers, shown in Supplementary File S1 Section D.

## 4 Discussion

### 4.1 Distributed representation of DNA strings using sparse representation

A key distinction between traditional and more recent approaches to motif discovery is in how motifs are represented during optimization. Traditional methods, such as STREME and HOMER, typically use local representations, such as PWMs, for motif characterization during optimization. In contrast, recent approaches often use deep learning that leverage distributive representations to learn and represent motifs (Hinton *et al.* 1986). The choice between the two types of representation involves a trade-off between interpretability and expressiveness, with local representations being easier to interpret. However, problems that characterize motifs with local representations has generally been shown to be NP-hard (Bafna *et al.* 1997, Li *et al.* 1999, Akutsu *et al.* 2000). Due to this, common heuristics, such as substring masking, are often used during optimization to find motifs, resulting a sequential procedure for motif discovery (Bailey and Elkan 1995, Heinz *et al.* 2010, Bailey 2021). This sequential procedure may pose challenges as primary motifs can overlap with secondary motifs, including gapped motifs and transposable elements present in the dataset. Our result demonstrates the existence of these secondary motifs, and there are simulated experiments indicate that methods such as STREME and HOMER are less effective when multiple motifs overlaps (Chu and Stormo 2022).

We select sparse representations for DNA strings as they provide a distributive representation with a clear interpretation. By approximating DNA strings as a sum of linear convolutions (equation (1)), the non-zero components in the sparse code essentially act as indicators, indicating where filters should be used to represent DNA substrings (Fig. 2A). The combined sparse code, which we refer to as code images, provides a high-level view on the spatial arrangements of the filters, which we build another sparse representation upon (Fig. 2B). This sparse representation on the code images enables us to identify the conserved patterns in the dataset, akin to enumerating k-mers at the nucleotide level. Yet, by enumerating at the image level, the spatial relationship in between the regulatory elements is preserved, in contrast to k-mers enumerations, which do not account for the spatial information.

### 4.2 Comparison to recent deep learning methodologies in regulatory genomics

As with recent work on deep learning for regulatory genomics (Alipanahi *et al.* 2015, Avsec *et al.* 2021b, Yuan and Kelley 2022), our approach use distributive representations to characterize patterns in DNA strings. Additionally, our method incorporates the design of a neural network (Fig. 3). However, our network design process is distinct from standard deep learning practices. Rather than using traditional design tools, we create the network architecture by unfolding the iterative algorithm detailed in Section 2.3. The unfolding technique incorporates hyperparameters, such as sparsity-inducing parameters, step-sizes, and penalty parameters from problem 2, as part of the network architecture, which are automatically tuned with backpropagation (Gregor and LeCun 2010, Monga *et al.* 2021). This results in a more expressive model compared with the original. We note that our network is fully interpretable as the forward pass can be seen as optimizing the objective posed by problem 2 via gradient descent. Inferring the motifs relies on enumerating at the image level (Section 2.5 and Fig. 2), without relying on explanation methods such as SHAP (Lundberg and Lee 2017). Our network is light in parameters (Section 2.7.1), allowing us to quickly train and infer motifs in a matter of minutes using a single GPU, and only requires DNA strings (FASTA files) as inputs.

### 4.3 Extensions to other regulatory genomics problems

Our method produces a computational graph (Fig. 3), which permits us to easily extend it to other regulatory genomics problems, such as DNA classifications or regressions. To accomplish this, one can treat the sparse code as an embedding, analogous to the embeddings constructed in the CNNs (Fig. 1). Furthermore, with the filters in problem 2 held fixed, the sparse code provides an alternative way of representing the binding sites. This alternative approach could be valuable for estimating the *recognition code*, e.g. designing C2H2 zinc-fingers with novel specificity (Gupta *et al.* 2014, Najafabadi *et al.* 2015, Aizenshtein-Gazit and Orenstein 2022, Ichikawa *et al.* 2023).

### 4.4 Future work

Our method effectively captures the gapped motifs (Figs 5 and 6), but highly varying spacers are common in these motifs. For instance, a gapped motif in BZIP MA0495.1 from JASPAR appears with 26 different spacer lengths (Supplementary File S1 Section D), resulting in numerous PWMs in our results. We intend to improve motif summarization and visualization in the future.

## 5 Conclusion

We present a motif discovery method that includes the discovery of long, gapped, or overlapping motifs. Our key concept, enumerating at the image level, enables a more extensive and diverse pattern identification in DNA strings compared with enumerating the k-mers. Our study demonstrates that the sparse representation is a highly interpretable modeling technique for DNA strings. This approach enables us to reveal that transposable elements and gapped motifs are common in ChIP-Seq datasets from JASPAR, Factorbook, and Remap (Castro-Mondragon *et al.* 2022, Hammal *et al.* 2022, Pratt *et al.* 2022). Our methodology is compatible with the deep learning paradigm through deep unfolding, enabling us to extend it to various regulatory genomics problems.

## Supplementary Material

btad378_Supplementary_Data
