# Supplementary material for: Finding motifs using DNA images derived from sparse representations
Source: Bioinformatics. 2023 Jun 9;39(6):btad378. doi: 10.1093/bioinformatics/btad378 (PMC10290554; doi:10.1093/bioinformatics/btad378)
Supplement: btad378_Supplementary_Data [file btad378_supplementary_data.zip › supplement.pdf]

Finding Motifs Using DNA Images Derived From Sparse Representations
(Supplemental Information)

Contents

A Acquiring Experimental Data from JASPAR, FactorBook, and ReMap **2**
- A.1 JASPAR 2
- A.2 Factorbook 2
- A.3 ReMap 2
- A.4 Avsec et al. 2

B Implementation **2**
- B.1 Programming language and packages 2
- B.2 Merge the PWMs using Average Log-Likelihood Ratios 2
- B.3 Estimating the length of each PWM 3
- B.4 Estimating the number of occurrences of each motif (PWM) 3
  - B.4.1 Approximate the score threshold for long motifs and long motifs that contain gaps . . 3

C Motif Representation: Choosing between Soft and Hard Clustering **4**
- C.1 Soft clustering representation 4
- C.2 Hard clustering representation 4
- C.3 Statistical significance of the motifs in both Soft and Hard clustering representations 5

D Variability in the Discovery of Gapped Motifs **6**

E Identification of Transposable Elements in JASPAR Datasets **7**

F Experimental results **10**

A Acquiring Experimental Data from JASPAR, FactorBook, and ReMap

A.1 JASPAR

Several datasets from JASPAR [Cas+22] have already included the fasta file used for motif discovery. For example, the dataset MA0495.1 has a FASTA file directly available for download in the Binding sites information section. We download the JASPAR profiles that has available FASTA file for download for classes of factors including BZIP, C2H2, MADS box factors, Paired box factors, Nuclear receptors with C4 zinc fingers, Fork head/winged helix factors, Tryptophan cluster factors, Other C4 zinc finger-type factors, Rel homology region (RHR) factors, and p53 domain factors. We use JASPAR 2022 version for this work. The JASPAR datasets require no further processing and contains the sites that used to estimate the primary motifs. Our methods takes in the FASTA file as input and treat it as unaligned DNA strings for motif discovery.

A.2 Factorbook

We obtained several transcription factor datasets (CTCF, Oct4, and STAT1) from Factorbook. For each dataset, we first located the center of each available peak and then extended 65 bp on both sides of the center to construct a new dataset for motif discovery.

A.3 ReMap

We selected several CTCF datasets from ReMap and used the peaks from each dataset for further analysis. We followed the same data processing steps as we did with the Factorbook datasets.

A.4 Avsec et al.

We obtained the ChIP-Nexus experiment's peak summit from `https://zenodo.org/record/3371216` [Avs+21]. Using the peak summits in Oct4, Sox2, Nanog, and Klf4, we again extended 65 bp on both sides of the peak summit to construct new datasets for motif discovery

B Implementation

B.1 Programming language and packages

We implement our method in the Julia programming language [Bez+17], available from the Julia registry via the command `add MOTIFs`. We use Flux.jl and Zygote.jl [Inn18; Inn+19] for the auto-differentiation on the computational graph in figure 3 in the main paper. We implement a customized kernel code for speeding up the greedy alignment using CUDA.jl [BFD18]. We render the PWM logo with Weblogo [Cro+04] and the rest of the visualizations with Makie.jl [DK21].

B.2 Merge the PWMs using Average Log-Likelihood Ratios

We compare and merge PWMs derived from the triplets using the average log-likelihood ratio (ALLR) [WS03]. Note that given two PWMs $\boldsymbol{P}_1$ and $\boldsymbol{P}_2$, their corresponding count matrices $\boldsymbol{C}_1, \boldsymbol{C}_2$ and frequency matrices $\boldsymbol{F}_1, \boldsymbol{F}_2$, and the assumed genomic background $\boldsymbol{B}_{\mathsf{A}}, \boldsymbol{B}_{\mathsf{C}}, \boldsymbol{B}_{\mathsf{G}}, \boldsymbol{B}_{\mathsf{T}}$, the ALLR of column i of $\boldsymbol{P}_1$ and column j for $\boldsymbol{P}_2$ is defined as

$$\frac{\sum_\alpha \boldsymbol{C}[\alpha,i]\log_2\left(\boldsymbol{F}[\alpha,j]/\boldsymbol{B}_\alpha\right) \; + \; \sum_\alpha \boldsymbol{C}[\alpha,j]\log_2\left(\boldsymbol{F}[\alpha,i]/\boldsymbol{B}_\alpha\right)}{\sum_\alpha \boldsymbol{C}[\alpha,i]+\boldsymbol{C}[\alpha,j]}. \tag{1}$$

The physical interpretation of ALLR is that it measures how two proteins binding to each other's binding sites [WS03]. A "good" ALLR value implies that two proteins accept each other's sites or that their sites are equivalent. The calculation of ALLR is computationally simple and the distribution of ALLR approximately an

extreme value distribution, which allows us to quickly determine whether the pair of PWMs is similar. In our implementation, we merge pairs of PWMs if their ALLR is more than 0.95. We merge such a pair by adding the corresponding two count matrices. If a pair of PWMs have different lengths, we can always check the shorter one's collection of DNA strings in the dataset to match the length of the longer one, allowing us to compare them.

B.3 Estimating the length of each PWM

We adopt the following simple procedure to estimate the length of the motifs: once we merge the PWMs (section B.2), we obtain a set of merged PWMs. We expand the width of the merged PWM if each merged PWM's respective MSA has neighboring sites that are not included in the MSA but have information content higher than 0.1 bit. Once we are done with expanding the width, we trim the width of a PWM of both ends if either end of the PWM has a column that has information content lower than 1 bit.

B.4 Estimating the number of occurrences of each motif (PWM)

To determine the number of occurrences of each motif represented by a PWM in the dataset, we search the DNA strings for positions that allowed for a PWM to score as high as the log-likelihood ratio score threshold, denoted as $s_{\boldsymbol{P}}$. We implemented pvalue2score (`https://github.com/kchu25/MotifPvalue.jl`), an approximation algorithm developed by Touzet and Varre [TV07], to determine the score threshold $s_{\boldsymbol{P}}$ for each PWM. The calculation for the score threshold $s_{\boldsymbol{P}}$ for each PWM $\boldsymbol{P}$ requires a specified p-value[1], which we set to be $1e{-}4$.

B.4.1 Approximate the score threshold for long motifs and long motifs that contain gaps

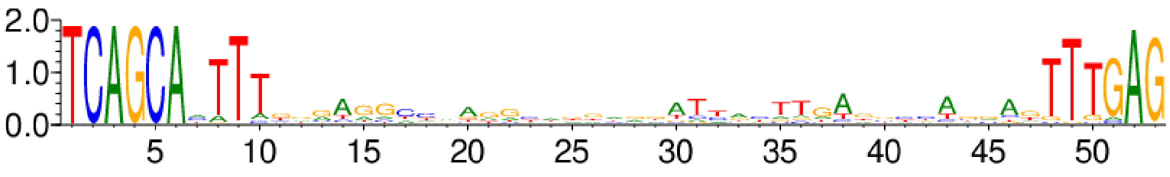

Figure 1: A long and gapped motif found in BZIP MA0150.2 from JASPAR [Cas+22]. The binding region, as defined in section B.4.1, consists of consecutive columns in a Position Weight Matrix (PWM) that have a high information content. Specifically, the left and right portions of the columns in this PWM correspond to the binding regions.

By default, we can simply calculate the score threshold for each Position Weight Matrix (PWM) and use it to estimate the number of occurrences. The number of occurrences of each PWM are then done by identifying the positions that gives a score above the score threshold. However, we have encountered several practical issues with this approach, such as:

1. The algorithm pvalue2score has exponential run time complexity that increases with the number of columns in the input PWM.
2. The estimates of score threshold for gapped motifs, i.e., PWMs with high information content columns separated by low information content columns, may be overly liberal, as they are more appropriately counted as the occurrence of multiple binding sites.

[1]The p-value in this context is the probablility of the background model, a product multinomial, to achieve a score at least as high as the score threshold s. Determine the score threshold for a PWM with a specified p-value is an NP-hard problem. For more details, see [TV07].

To address these concerns, we have implemented the following modifications. We first smooth out the information content of the PWMs by using the following Julia code to calculate the moving average to obtain a *smoothed information content* of each PWM:

```julia
function moving_average(A::AbstractArray, m::Int)
    out = similar(A)
    R = CartesianIndices(A)
    Ifirst, Ilast = first(R), last(R)
    I1 = m÷2 * oneunit(Ifirst)
    for I in R
        n, s = 0, zero(eltype(out))
        for J in max(Ifirst, I-I1):min(Ilast, I+I1)
            s += A[J]
            n += 1
        end
        out[I] = s/n
    end
    return out
end
```

where the input m in the above code is the window length. In all of our experiments, we set $m = 3$. We then define the *binding regions* of a PWM: a binding region in a PWM is a group of one or more consecutive columns that have smoothed information content larger than 0.5 bits. Once this is done, we calculate the score threshold for a PWM $\boldsymbol{P}$ by summing the score threshold calculated on each binding region of $\boldsymbol{P}$. This estimation results in a more conservative estimate of the score threshold of gapped motifs.

For long binding regions, i.e., more than 15 consecutive columns, we set the score threshold of such region as zero. This is a reasonable approach as the average score threshold for a PWMs with larger than 15 columns tend to be below zero with input p-value to pvalue2score set to $1e{-}4$.

C Motif Representation: Choosing between Soft and Hard Clustering

We use position weight matrices (PWMs) to represent the motifs in our results. We separate the motifs representation into two kinds of representations:

- Soft clustering representation
- Hard clustering representation

C.1 Soft clustering representation

Recall that a PWM represents the average binding pattern of a set of binding sites. Therefore, similar to soft clustering models such as Gaussian mixtures, the binding sites of each PWM may overlap with those of others. To address this, we adopt a soft clustering approach where we allow the multiple sequence alignment (MSA) of each PWM to use DNA substrings found in other PWMs.

C.2 Hard clustering representation

If we want to avoid the potential overlaps between binding sites of different PWMs in the soft clustering representation, we can re-estimate each PWM using mutually exclusive DNA strings. To do so, for each DNA string s in the multiple sequence alignment (MSA) of a PWM P, we only use s to estimate P if P has the highest log-likelihood ratio score[Sto00] compared to all other PWMs that also overlaps s. This ensures that no two motifs can occur in the same site, preventing potential conflicts in motif assignments.

C.3 Statistical significance of the motifs in both Soft and Hard clustering representations

The primary trade-off between the soft and hard clustering representations of motifs is the method used to calculate statistical significance. Recall that we estimate the statistical significance of each motif using Fisher exact test (main paper section 2.7.2). The Fisher exact test quantifies how enriched a motif is relative to the genomic background. If a motif is statistically significant (i.e., a low p-value), it means that it is occurring a lot more in the foreground (the dataset) than in the genomic background, and vice versa.

We estimate the p-values from Fisher exact test on a test set: a set of DNA strings that is left completely untouched during the training phase of our method, and a corresponding control set that shuffles each string in the test set that preserves the frequency of 2-mers (https://github.com/kchu25/SeqShuffle.jl). We construct the test set by randomly selecting 15% of the data from the input dataset.

A soft clustering representation gives a more lenient estimate of the statistical significance of a motif. A hard clustering representation, by contrast, is more conservative, because by construction, the motifs cannot occur simultaneously at the same sites. Our findings suggest that the hard clustering representation may be overly conservative. This is because in this representation, many PWMs (motifs) may fail to demonstrate enrichment in the test set, often due to the mutual exclusivity of the MSAs for each PWM.

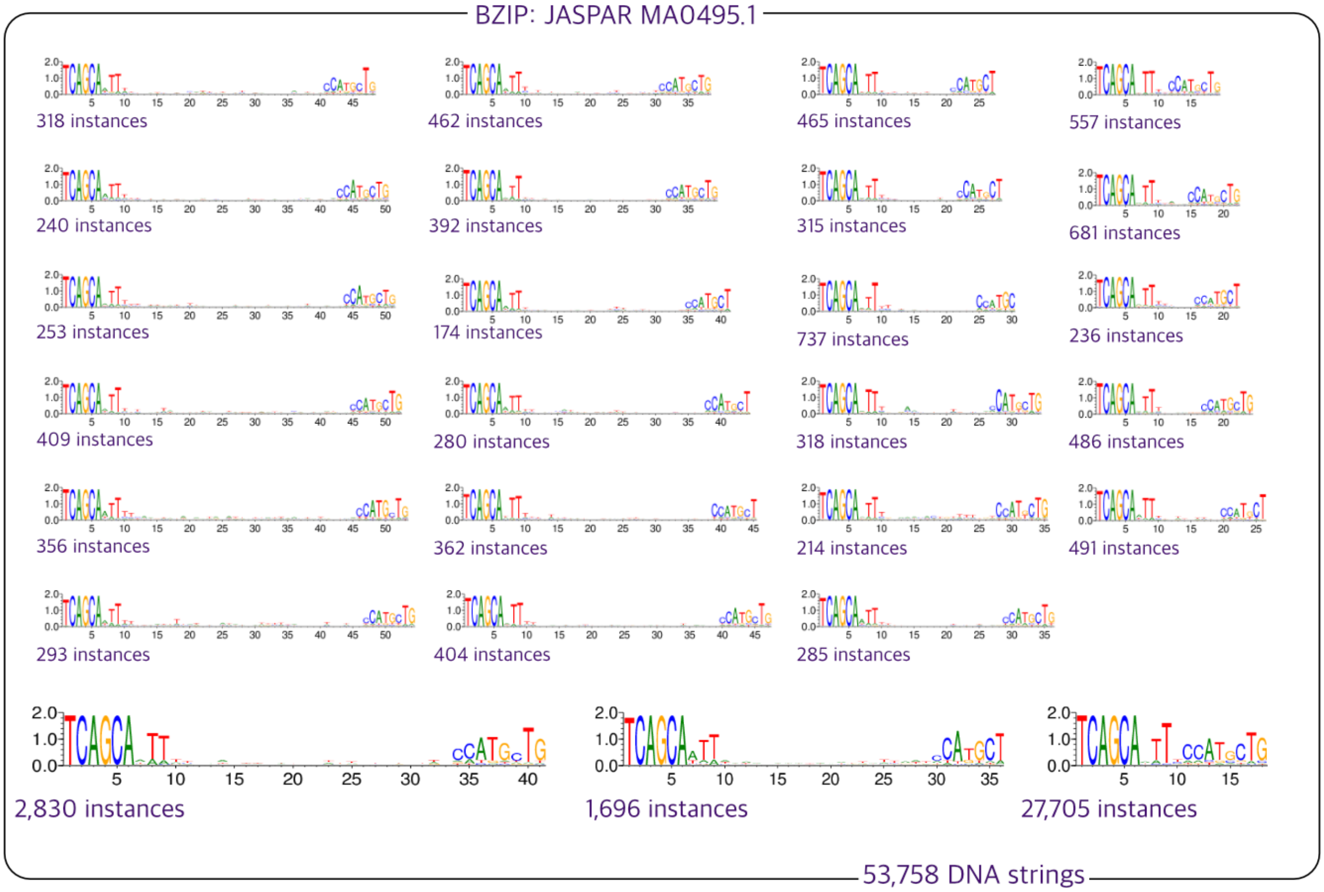

Figure 2: A gapped motif discovered from the (TAFF factor) BZIP MA0495.1 dataset from JASPAR [Cas+22]. The primary motif co-occurs with its partial complement with 26 spacers, and there are 3 spacers within such pattern in the dataset that are more enriched than the rest.

D Variability in the Discovery of Gapped Motifs

The number of motifs discovered from our method could be large and varied. For example, in BZIP MA0495.1 dataset from JASPAR [Cas+22], we find that the primary motif `TCAGCA` co-occurs with its partial complement `TGCTG` with a 26 spacers, shown in figure 2. Our method, currently, is primarily designed to find and quantify all such variabilities that may occur in the dataset. We intend to improve motif summarization and visualization in the future.

E Identification of Transposable Elements in JASPAR Datasets

We discovered that transposable elements are common in ChIP-Seq datasets, occurring in 50 out of the 91 ChIP-Seq datasets we examined from JASPAR [Cas+22]. The occurrence of transposable elements in these datasets is typically sparse, with only a low number of instances observed. Further, we found that there could be more than one transposable elements in these datasets. Below, we show just a single example of a tranposable element that we found for each datasets that contain transposable elements. The full results is in supplementary file 2. All of the transposable elements presented here are identified using Dfam [Hub+16], and we indicate the group to which each transposable element belongs in the TE column.

Table 1: Trasposable elements found from JASPAR

JASPAR ID	protein	instances/# strings	motif	TE	Species
MA0005.2	MADS Box factors	33/1,473	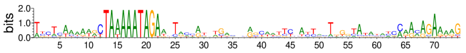	L1	Homo Sapiens
MA0083.2	MADS Box factors	21/2,277	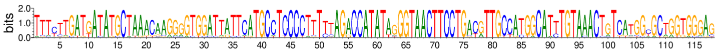	ERV1	Homo Sapiens
MA0497.1	MADS Box factors	19/2,209	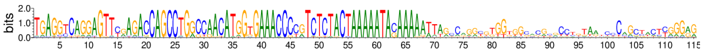	Alu	Homo Sapiens
MA0102.3	BZIP	15/15,318	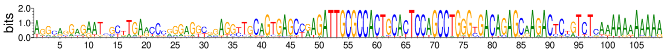	Alu	Homo Sapiens
MA0150.2	BZIP	17/726	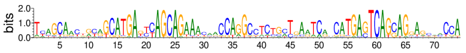	ERV2	Homo Sapiens
MA0462.1	BZIP	72/10,522	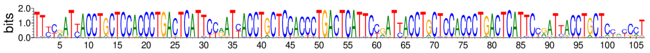	ERV1	Homo Sapiens
MA0476.1	BZIP	115/29,396	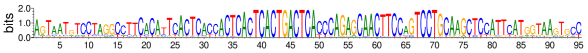	Tiggers	Homo Sapiens
MA0477.1	BZIP	36/5,272	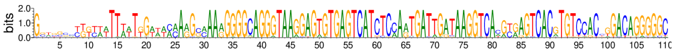	ERV2	Homo Sapiens
MA0478.1	BZIP	15/5,318	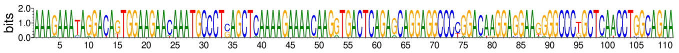	ERV1	Homo Sapiens
MA0488.1	BZIP	259/20,968	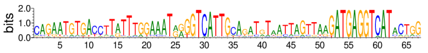	MaLR	Homo Sapiens
MA0489.1	BZIP	78/10,956	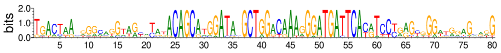	Tiggers	Homo Sapiens
MA0490.1	BZIP	67/16,992	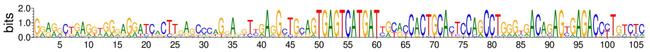	Alu	Homo Sapiens
MA0491.1	BZIP	3,188/38,710	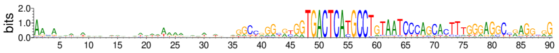	Alu	Homo Sapiens
MA0492.1	BZIP	316/33,631	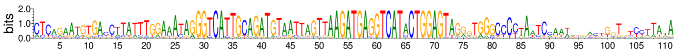	MaLR	Homo Sapiens
MA0495.1	BZIP	377/53,758	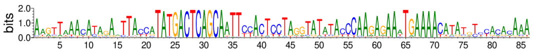	ERV1	Homo Sapiens
MA0496.1	BZIP	494/60,790	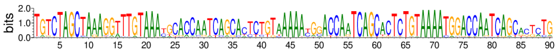	ERV1	Homo Sapiens

Table 2: Trasposable elements found from JASPAR - continue

JASPAR ID	protein	instances/# strings	motif	TE	Species
MA0501.1	BZIP	12/1,090	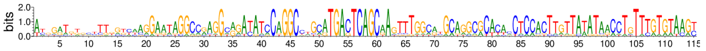	ERV3	Homo Sapiens
MA0506.1	BZIP	17/4,624	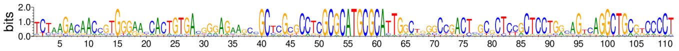	SST1	Homo Sapiens
MA0079.1	C2H2	28/8,734	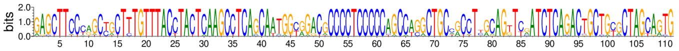	L1	Homo Sapiens
MA0095.2	C2H2	129/7,171	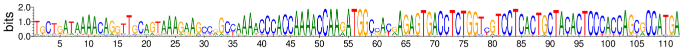	ERV1	Homo Sapiens
MA0493.1	C2H2	15/525	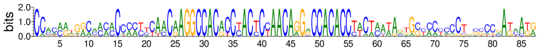	MaLR	Mus musculus
MA0527.1	C2H2	18/705	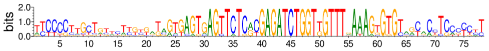	MaLR	Homo Sapiens
MA0599.1	C2H2	21/13,610	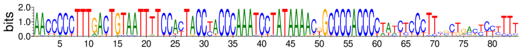	ERV1	Homo Sapiens
MA0148.3	Fork-head	53/22,008	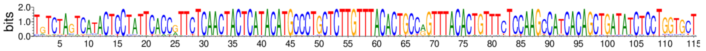	ERV1	Homo Sapiens
MA0471.1	Fork-head	15/2,757	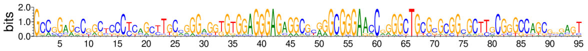	ERV1	Homo Sapiens
MA0479.1	Fork-head	38/8,211	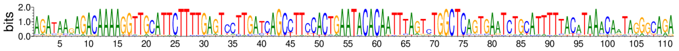	ERV1	Homo Sapiens
MA0481.1	Fork-head	25/311	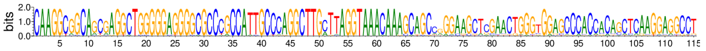	L1	Homo Sapiens
MA0510.1	Fork-head	58/3868	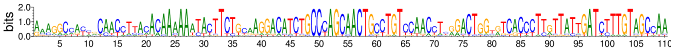	ERV1	Homo Sapiens
MA0593.1	Fork-head	14/766	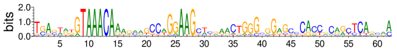	L1	Homo Sapiens
MA0600.1	Fork-head	26/2,345	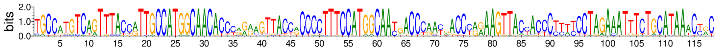	ERV1	Homo Sapiens
MA0007.2	nuclear receptor C4	26/11,206	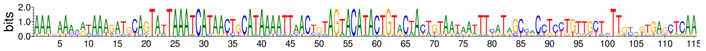	Tigger	Homo Sapiens
MA0114.2	nuclear receptor C4	17/16,768	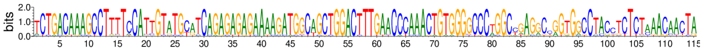	ERV1	Homo Sapiens
MA0258.2	nuclear receptor C4	149/8243	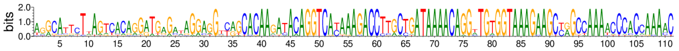	ERV1	Homo Sapiens
MA0484.1	nuclear receptor C4	23/9452	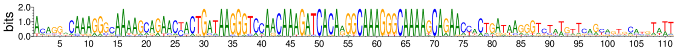	ERV2	Homo Sapiens
MA0505.1	nuclear receptor C4	32/1,702	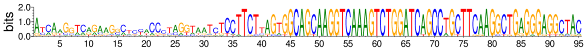	ERV2	Homo Sapiens

Table 3: Trasposable elements found from JASPAR - continue

JASPAR ID	protein	instances/# strings	motif	TE	Species
MA0036.2	Other C4 ZF	29/4,380	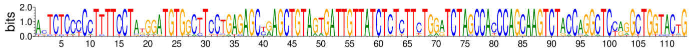	L1	Homo Sapiens
MA0140.2	Other C4 ZF	603/4,954	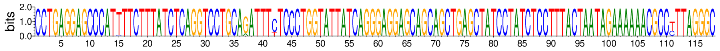	ERV2	Homo Sapiens
MA0106.2	P53 factor	53/1,231	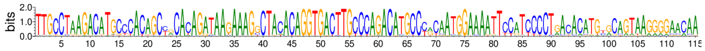	ERV1	Homo Sapiens
MA0525.1	P53 factor	250/9,362	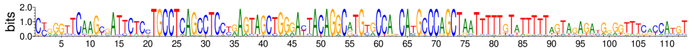	Alu	Homo Sapiens
MA0014.2	P53 factor	22/896	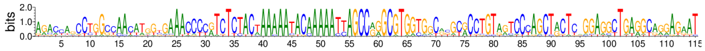	Alu	Homo Sapiens
MA0105.3	Rel homology region	22/5,112	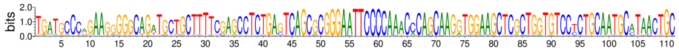	ERV1	Homo Sapiens
MA0154.2	Rel homology region	308/33853	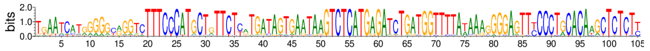	MaLR	Homo Sapiens
MA0137.3	STAT	363/3,629	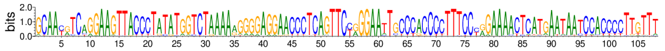	ERV1	Homo Sapiens
MA0144.2	STAT	355/21,620	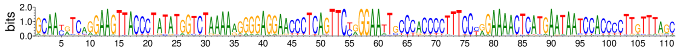	ERV1	Homo Sapiens
MA0517.1	STAT	26/620	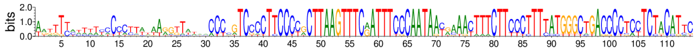	ERV1	Homo Sapiens
MA0518.1	STAT	28/2,873	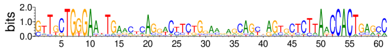	L2-end	Homo Sapiens
MA0519.1	STAT	20/16,507	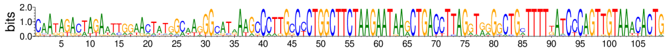	ERV2	Homo Sapiens
MA0050.2	Tryptophan cluster	15/1,362	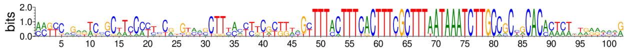	ERV1	Homo Sapiens
MA0100.2	Tryptophan cluster	54/979	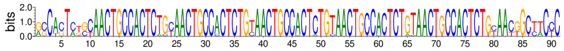	ERV2	Mus musculus
MA0544.1	Tryptophan cluster	17/310	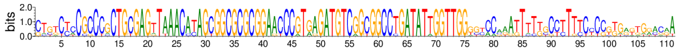	TC1	Caenorhabditis elegans

F Experimental results

Results on experimental data can be found at https://zenodo.org/record/7783033.

References

[Avs+21] Ziga Avsec et al. “Base-resolution models of transcription-factor binding reveal soft motif syntax”. In: *Nature Genetics* 53.3 (2021), pp. 354–366.

[Bez+17] Jeff Bezanson et al. “Julia: A fresh approach to numerical computing”. In: *SIAM review* 59.1 (2017), pp. 65–98.

[BFD18] Tim Besard, Christophe Foket, and Bjorn De Sutter. “Effective extensible programming: unleashing Julia on GPUs”. In: *IEEE Transactions on Parallel and Distributed Systems* 30.4 (2018), pp. 827–841.

[Cas+22] Jaime A Castro-Mondragon et al. “JASPAR 2022: the 9th release of the open-access database of transcription factor binding profiles”. In: *Nucleic acids research* 50.D1 (2022), pp. D165–D173.

[Cro+04] Gavin E Crooks et al. “WebLogo: a sequence logo generator”. In: *Genome research* 14.6 (2004), pp. 1188–1190.

[DK21] Simon Danisch and Julius Krumbiegel. “Makie. jl: Flexible high-performance data visualization for Julia”. In: *Journal of Open Source Software* 6.65 (2021), p. 3349.

[Hub+16] Robert Hubley et al. “The Dfam database of repetitive DNA families”. In: *Nucleic acids research* 44.D1 (2016), pp. D81–D89.

[Inn+19] Mike Innes et al. “A differentiable programming system to bridge machine learning and scientific computing”. In: *arXiv preprint arXiv:1907.07587* (2019).

[Inn18] Mike Innes. “Flux: Elegant machine learning with Julia”. In: *Journal of Open Source Software* 3.25 (2018), p. 602.

[Sto00] Gary D Stormo. “DNA binding sites: representation and discovery”. In: *Bioinformatics* 16.1 (2000), pp. 16–23.

[TV07] Hélène Touzet and Jean-Stéphane Varré. “Efficient and accurate P-value computation for Position Weight Matrices”. In: *Algorithms for Molecular Biology* 2.1 (2007), pp. 1–12.

[WS03] Ting Wang and Gary D Stormo. “Combining phylogenetic data with co-regulated genes to identify regulatory motifs”. In: *Bioinformatics* 19.18 (2003), pp. 2369–2380.
