# Supplementary figures and images for: Finding motifs using DNA images derived from sparse representations
Source: Bioinformatics. 2023 Jun 9;39(6):btad378. doi: 10.1093/bioinformatics/btad378 (PMC10290554; doi:10.1093/bioinformatics/btad378)

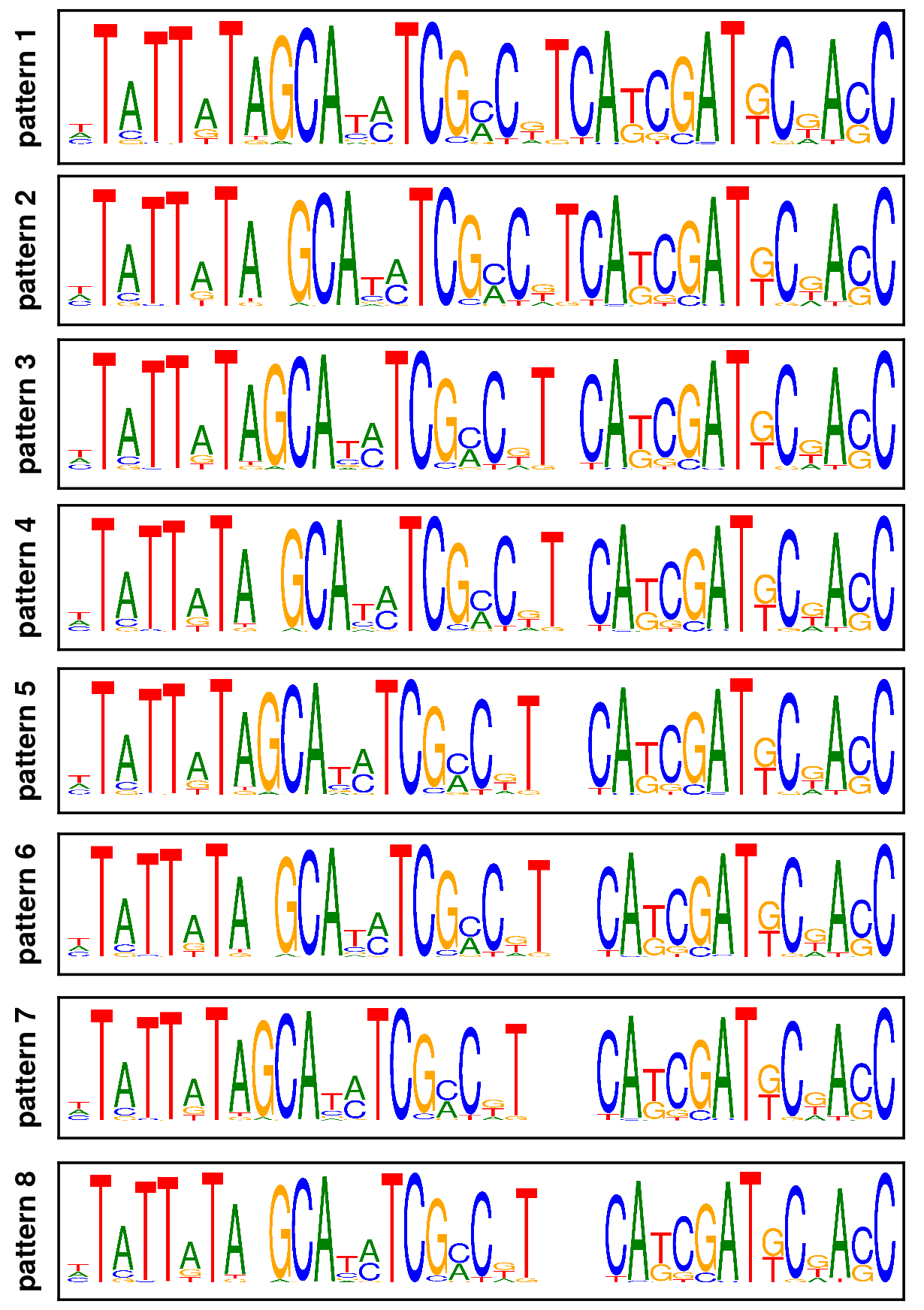

Supplement: btad378_Supplementary_Data [file btad378_supplementary_data.zip › supp_updated_chu/supp/supp_figures/sim_data_gap.png]

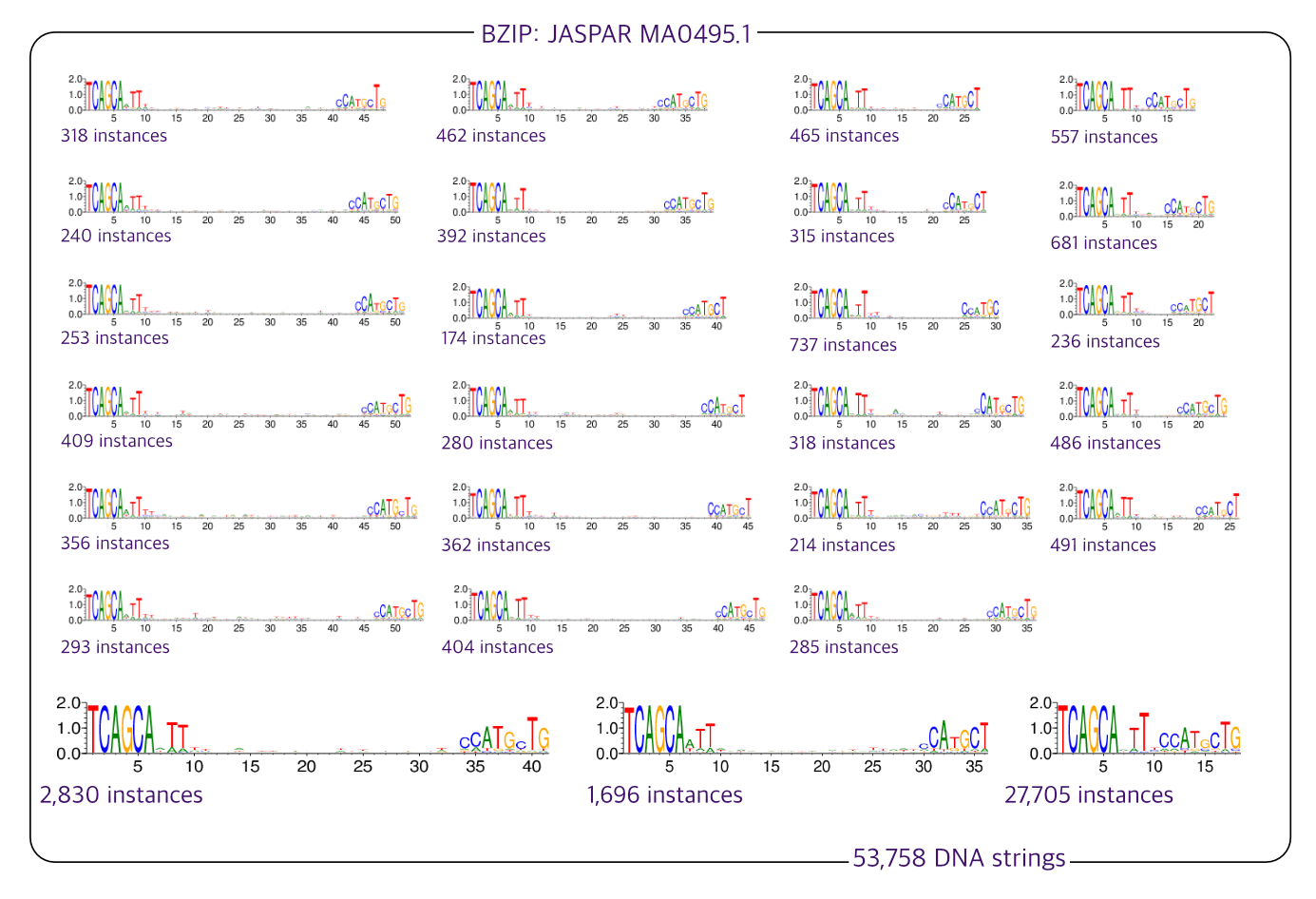

Supplement: btad378_Supplementary_Data [file btad378_supplementary_data.zip › supp_updated_chu/supp/supp_figures/gaps.png]

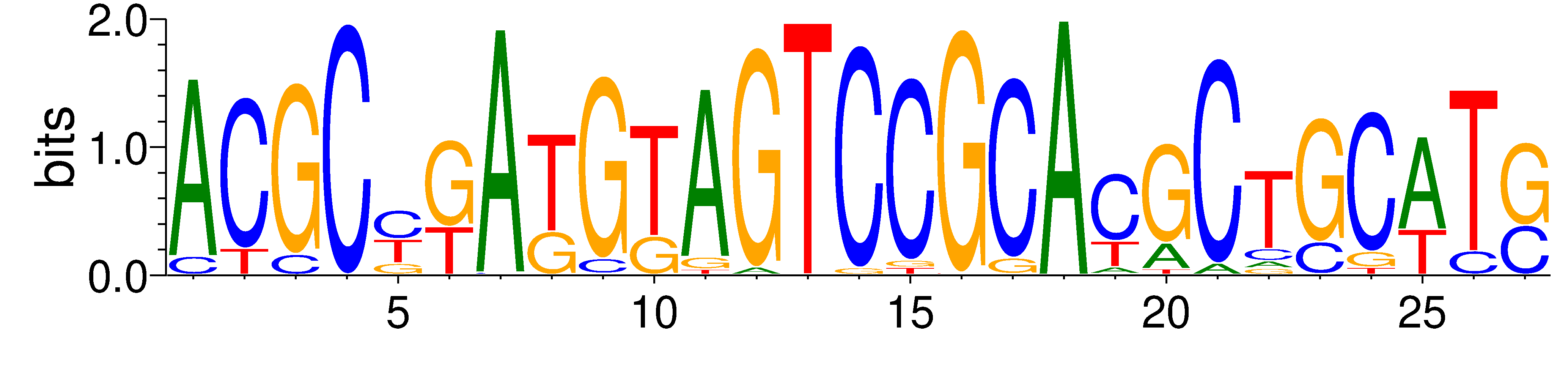

Supplement: btad378_Supplementary_Data [file btad378_supplementary_data.zip › supp_updated_chu/supp/supp_figures/example_sim.png]

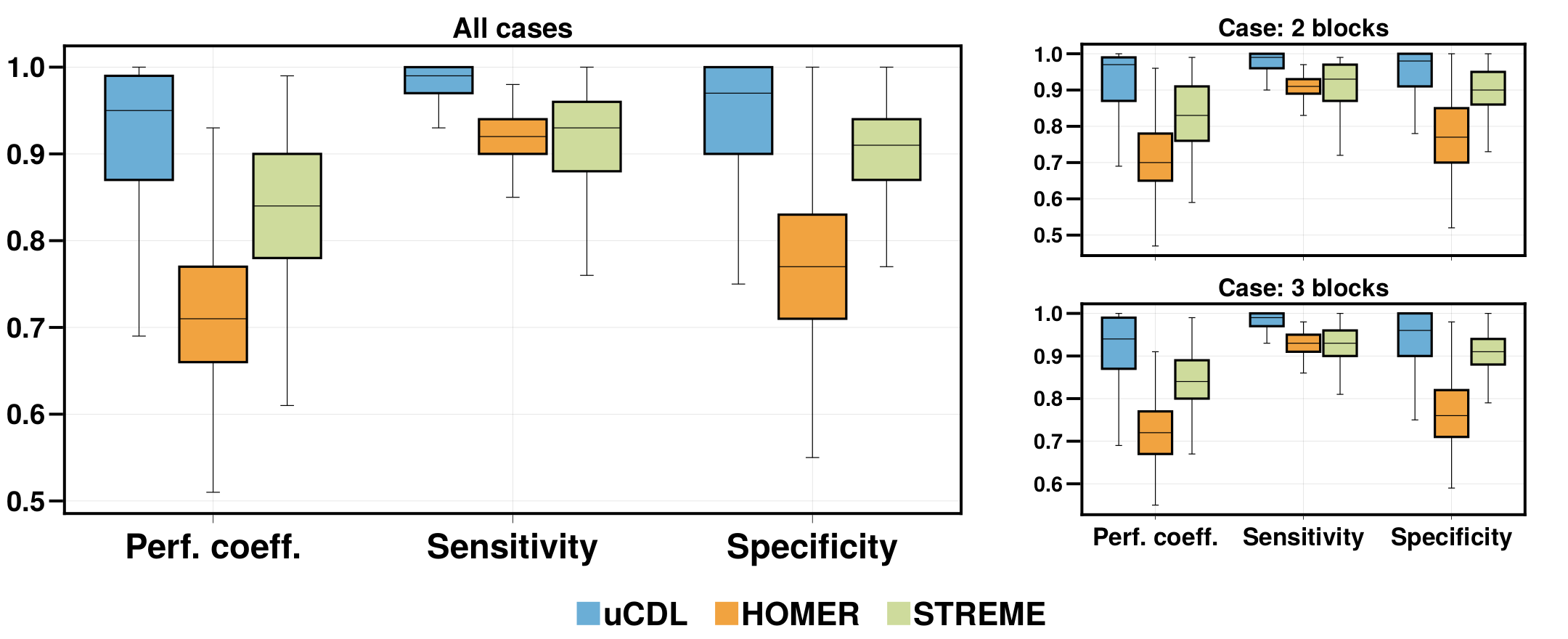

Supplement: btad378_Supplementary_Data [file btad378_supplementary_data.zip › supp_updated_chu/supp/supp_figures/perf_gap0.6.png]

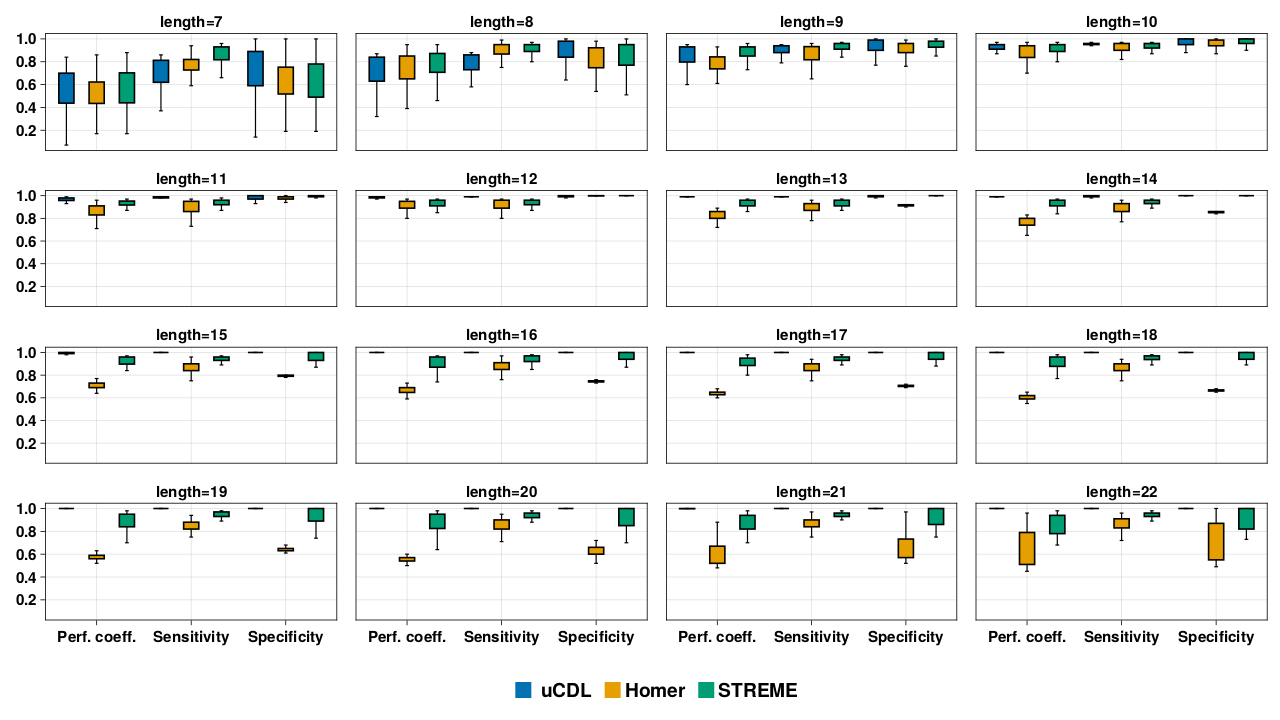

Supplement: btad378_Supplementary_Data [file btad378_supplementary_data.zip › supp_updated_chu/supp/supp_figures/perf_single.png]

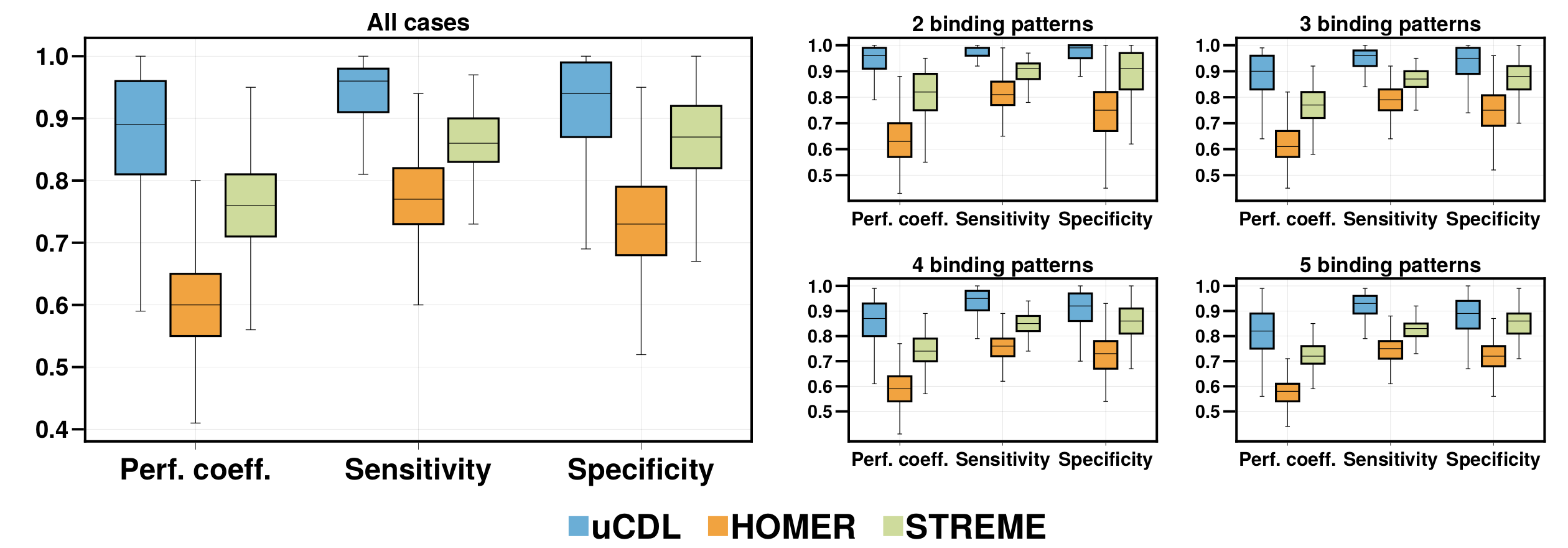

Supplement: btad378_Supplementary_Data [file btad378_supplementary_data.zip › supp_updated_chu/supp/supp_figures/perf_overall.png]

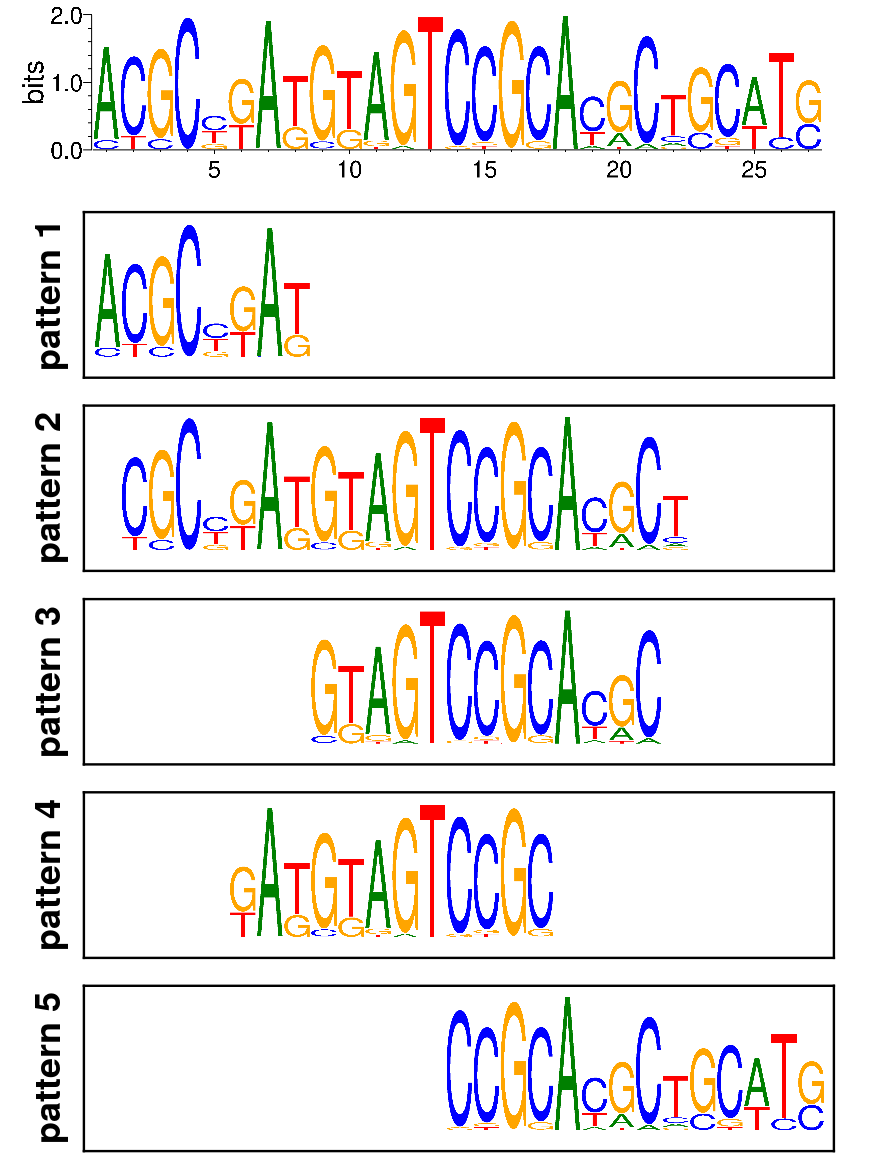

Supplement: btad378_Supplementary_Data [file btad378_supplementary_data.zip › supp_updated_chu/supp/supp_figures/sim_data.png]

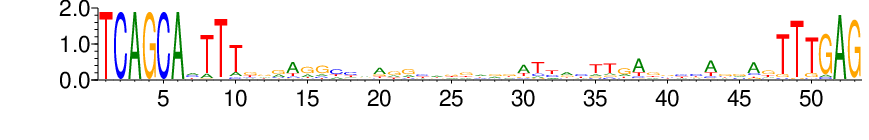

Supplement: btad378_Supplementary_Data [file btad378_supplementary_data.zip › supp_updated_chu/supp/supp_figures/a_gapped_motif.png]

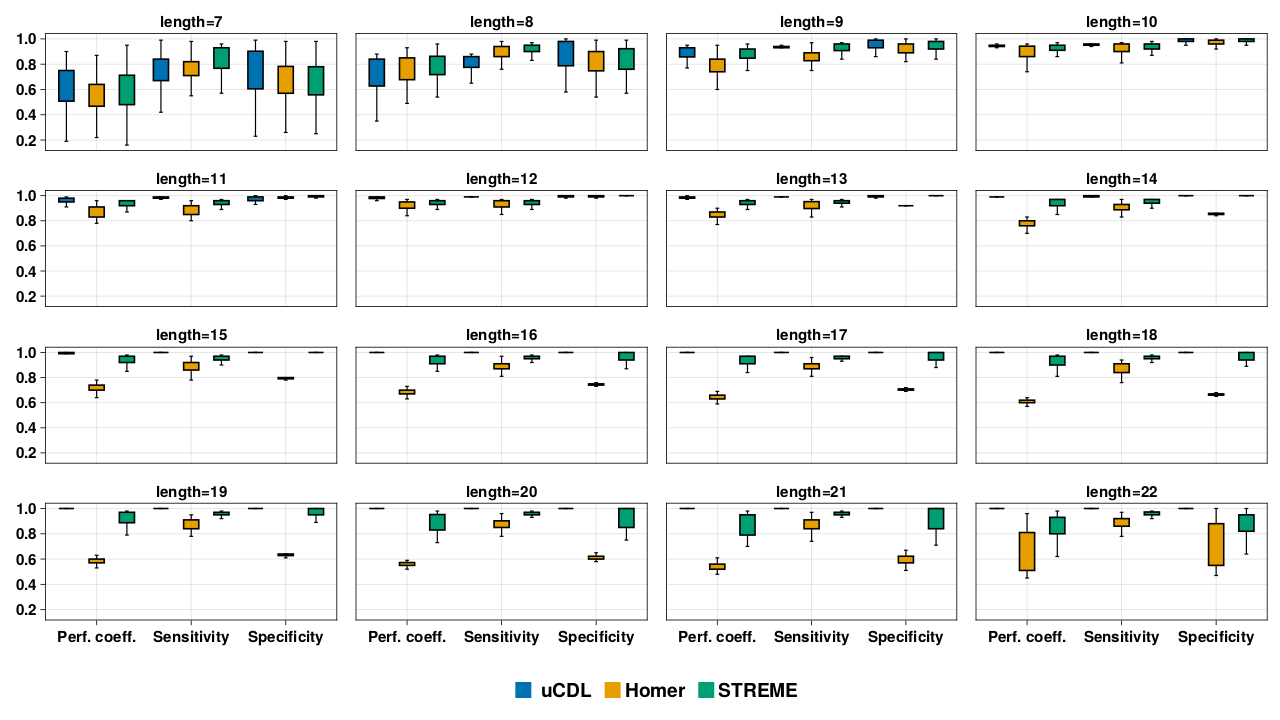

Supplement: btad378_Supplementary_Data [file btad378_supplementary_data.zip › supp_updated_chu/supp/supp_figures/perf_single0.6.png]

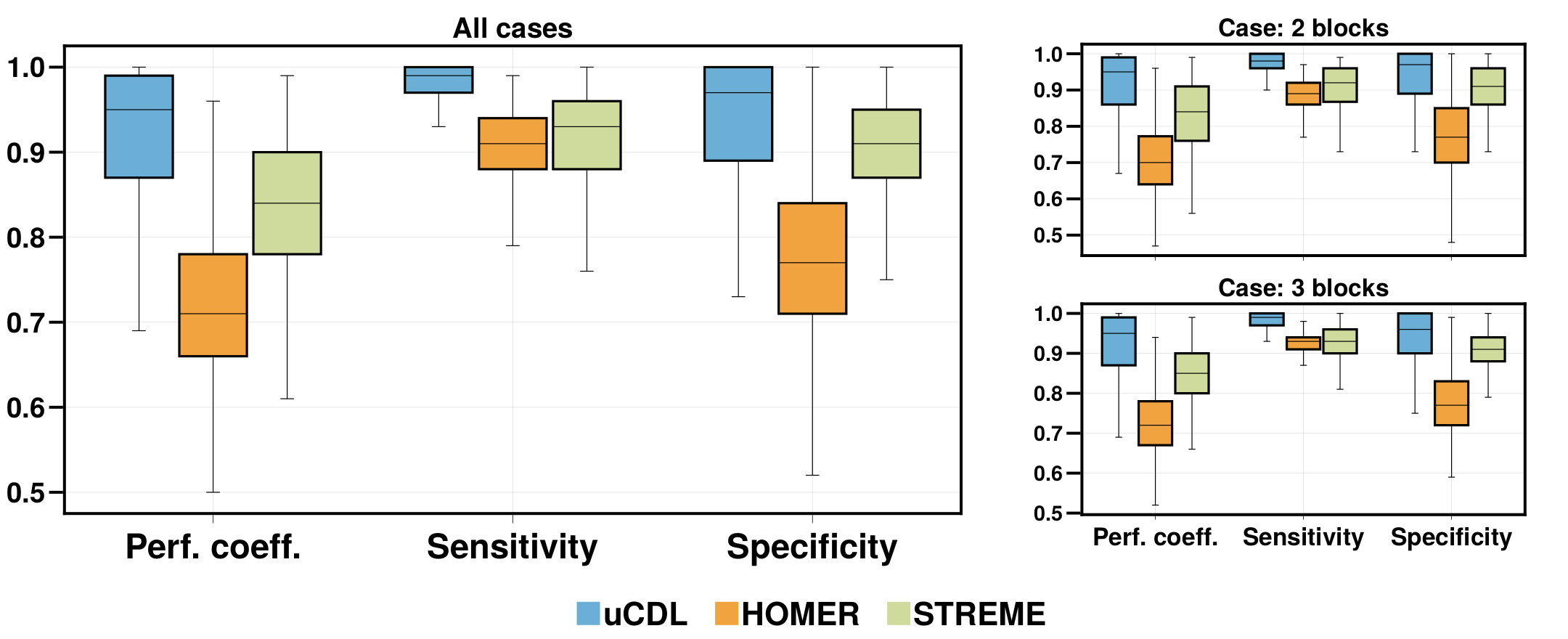

Supplement: btad378_Supplementary_Data [file btad378_supplementary_data.zip › supp_updated_chu/supp/supp_figures/perf_gap.png]

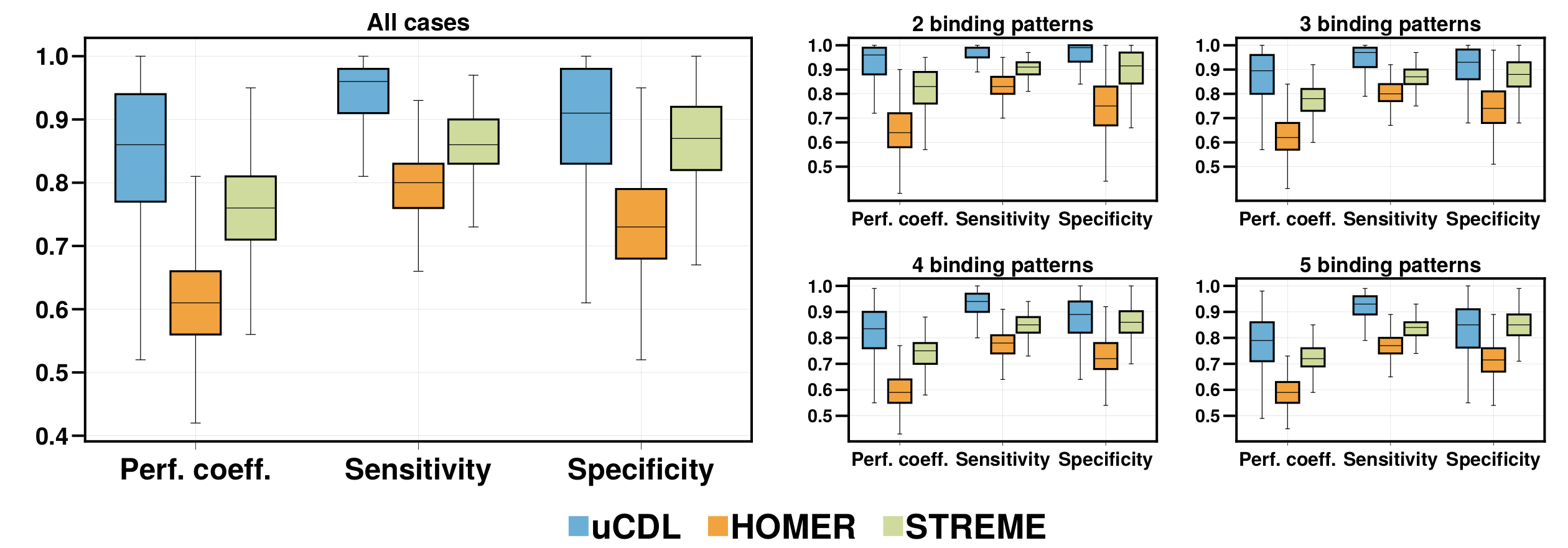

Supplement: btad378_Supplementary_Data [file btad378_supplementary_data.zip › supp_updated_chu/supp/supp_figures/perf_overall0.6.png]

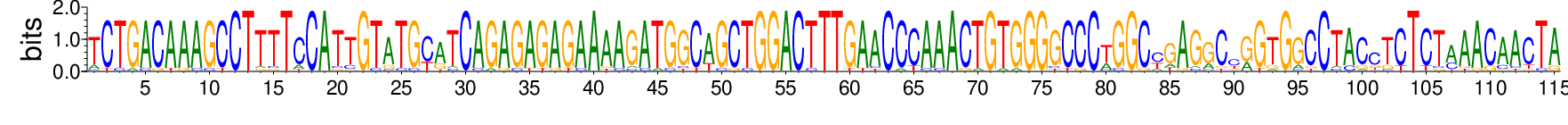

Supplement: btad378_Supplementary_Data [file btad378_supplementary_data.zip › supp_updated_chu/supp/supp_figures/tes/MA0114.2.png]

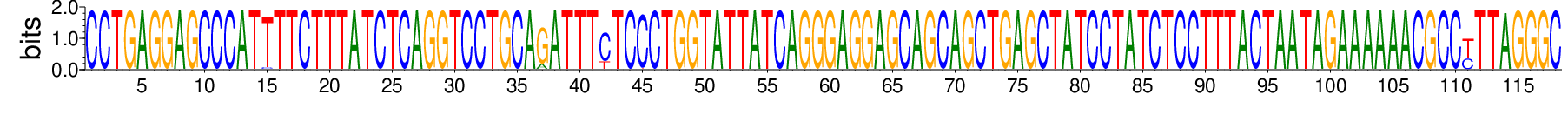

Supplement: btad378_Supplementary_Data [file btad378_supplementary_data.zip › supp_updated_chu/supp/supp_figures/tes/MA0140.2.png]

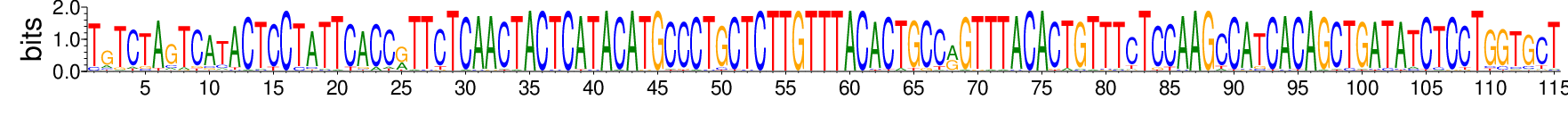

Supplement: btad378_Supplementary_Data [file btad378_supplementary_data.zip › supp_updated_chu/supp/supp_figures/tes/MA0148.3.png]

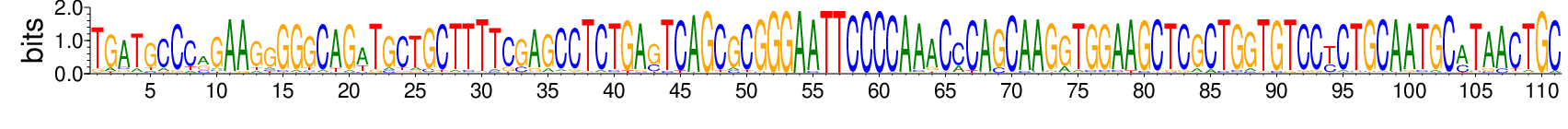

Supplement: btad378_Supplementary_Data [file btad378_supplementary_data.zip › supp_updated_chu/supp/supp_figures/tes/MA0105.3.png]

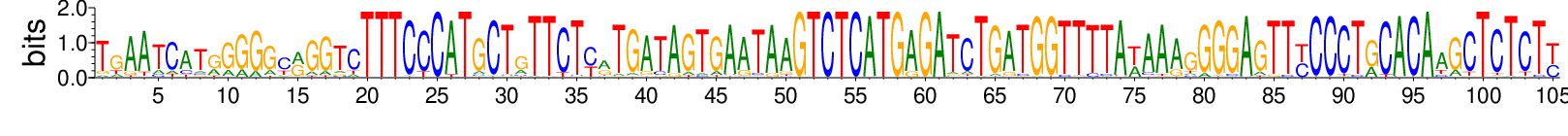

Supplement: btad378_Supplementary_Data [file btad378_supplementary_data.zip › supp_updated_chu/supp/supp_figures/tes/MA0154.2.png]

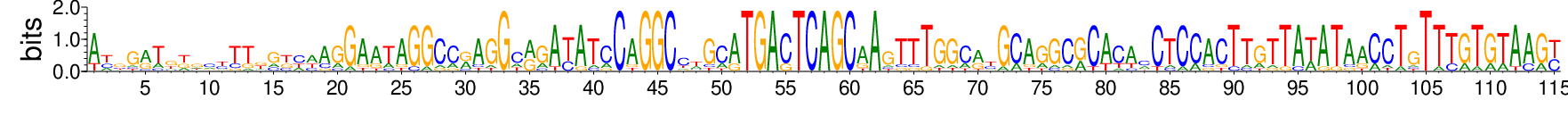

Supplement: btad378_Supplementary_Data [file btad378_supplementary_data.zip › supp_updated_chu/supp/supp_figures/tes/MA0501.1.png]

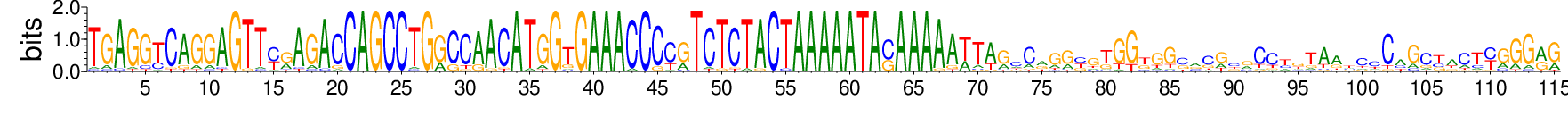

Supplement: btad378_Supplementary_Data [file btad378_supplementary_data.zip › supp_updated_chu/supp/supp_figures/tes/MA0497.1.png]

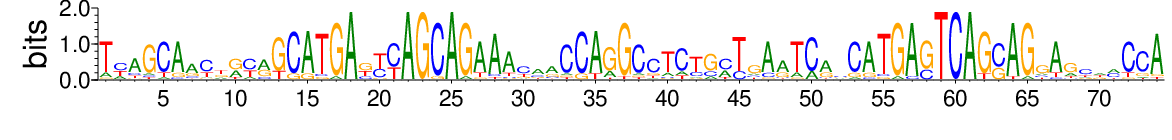

Supplement: btad378_Supplementary_Data [file btad378_supplementary_data.zip › supp_updated_chu/supp/supp_figures/tes/MA0150.2.png]

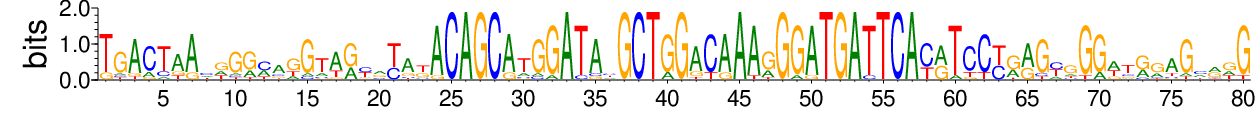

Supplement: btad378_Supplementary_Data [file btad378_supplementary_data.zip › supp_updated_chu/supp/supp_figures/tes/MA0489.1.png]

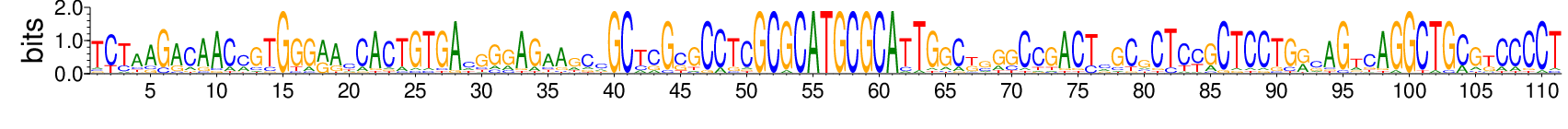

Supplement: btad378_Supplementary_Data [file btad378_supplementary_data.zip › supp_updated_chu/supp/supp_figures/tes/MA0506.1.png]

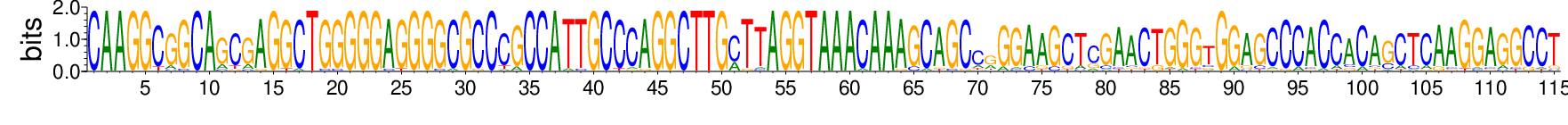

Supplement: btad378_Supplementary_Data [file btad378_supplementary_data.zip › supp_updated_chu/supp/supp_figures/tes/MA0481.1.png]

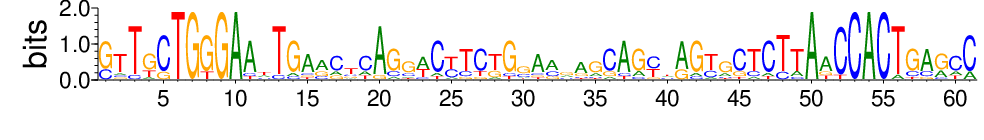

Supplement: btad378_Supplementary_Data [file btad378_supplementary_data.zip › supp_updated_chu/supp/supp_figures/tes/MA0518.1.png]

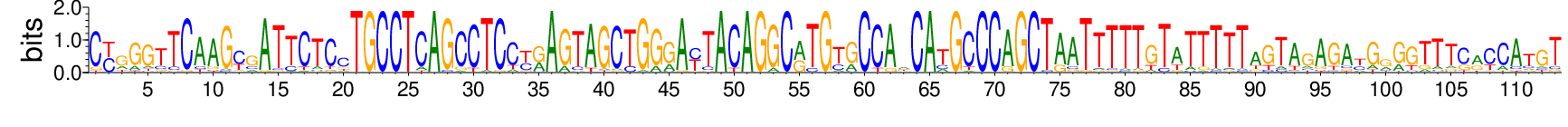

Supplement: btad378_Supplementary_Data [file btad378_supplementary_data.zip › supp_updated_chu/supp/supp_figures/tes/MA0525.1.png]

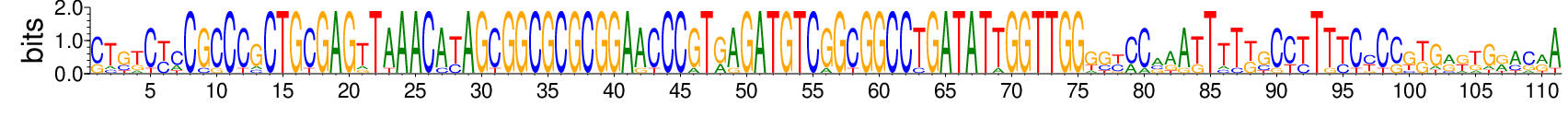

Supplement: btad378_Supplementary_Data [file btad378_supplementary_data.zip › supp_updated_chu/supp/supp_figures/tes/MA0544.1.png]

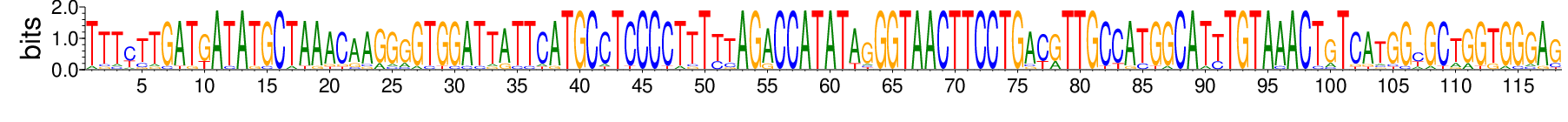

Supplement: btad378_Supplementary_Data [file btad378_supplementary_data.zip › supp_updated_chu/supp/supp_figures/tes/MA0083.2.png]

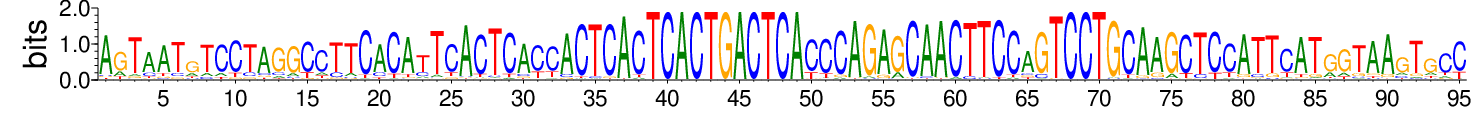

Supplement: btad378_Supplementary_Data [file btad378_supplementary_data.zip › supp_updated_chu/supp/supp_figures/tes/MA0476.1.png]

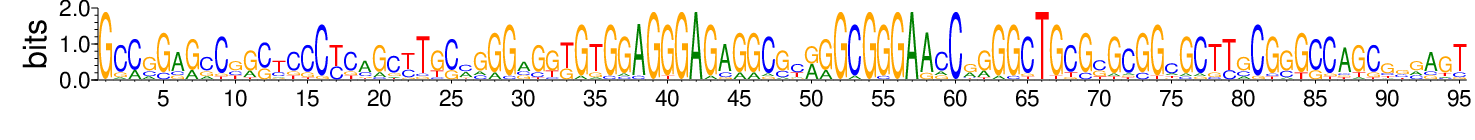

Supplement: btad378_Supplementary_Data [file btad378_supplementary_data.zip › supp_updated_chu/supp/supp_figures/tes/MA0471.1.png]

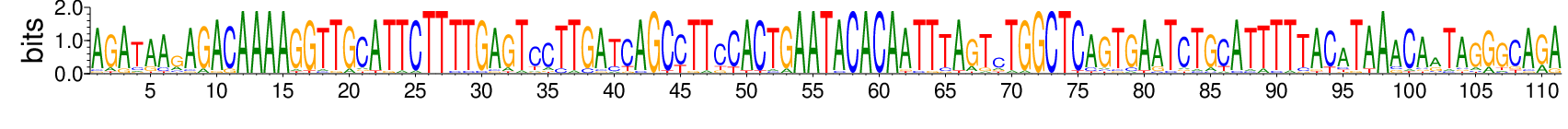

Supplement: btad378_Supplementary_Data [file btad378_supplementary_data.zip › supp_updated_chu/supp/supp_figures/tes/MA0479.1.png]

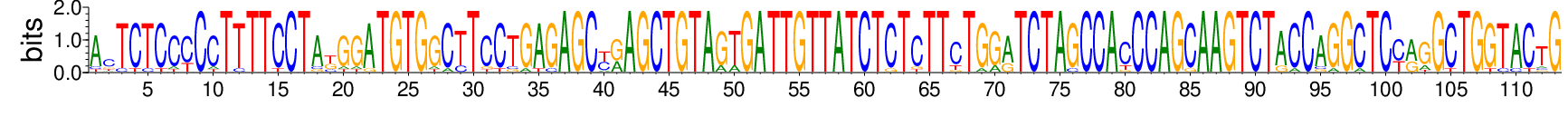

Supplement: btad378_Supplementary_Data [file btad378_supplementary_data.zip › supp_updated_chu/supp/supp_figures/tes/MA0036.2.png]

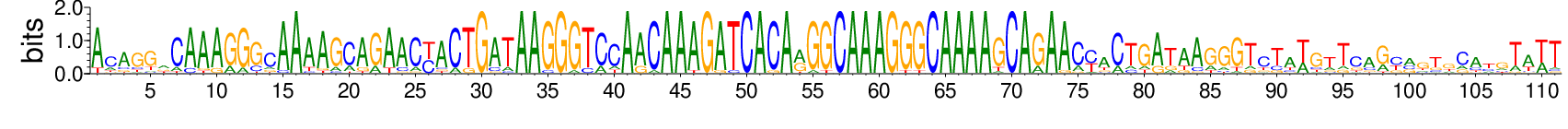

Supplement: btad378_Supplementary_Data [file btad378_supplementary_data.zip › supp_updated_chu/supp/supp_figures/tes/MA0484.1.png]

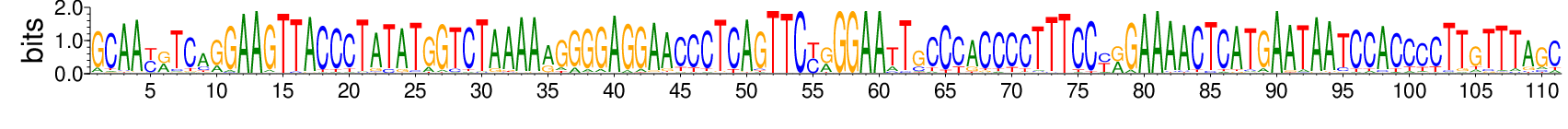

Supplement: btad378_Supplementary_Data [file btad378_supplementary_data.zip › supp_updated_chu/supp/supp_figures/tes/MA0144.2.png]

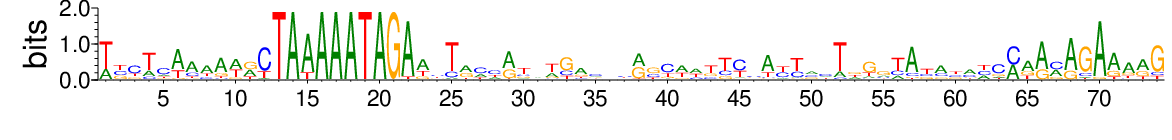

Supplement: btad378_Supplementary_Data [file btad378_supplementary_data.zip › supp_updated_chu/supp/supp_figures/tes/MA0052.2.png]

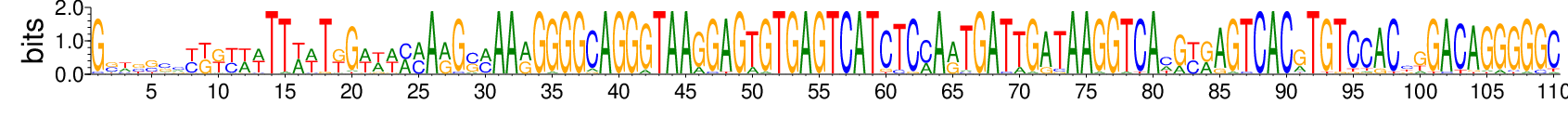

Supplement: btad378_Supplementary_Data [file btad378_supplementary_data.zip › supp_updated_chu/supp/supp_figures/tes/MA0477.1.png]

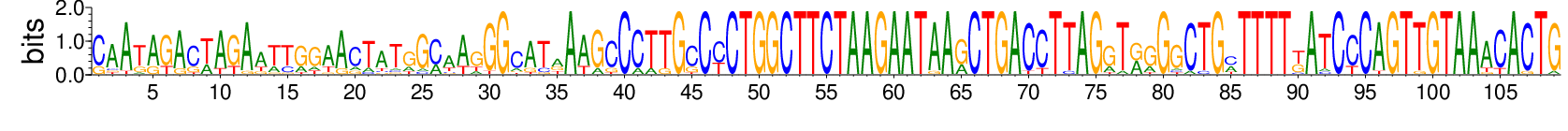

Supplement: btad378_Supplementary_Data [file btad378_supplementary_data.zip › supp_updated_chu/supp/supp_figures/tes/MA0519.1.png]

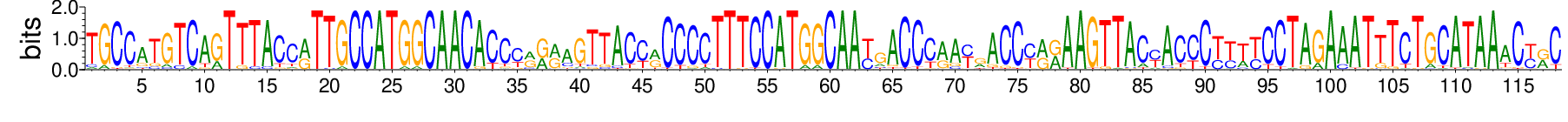

Supplement: btad378_Supplementary_Data [file btad378_supplementary_data.zip › supp_updated_chu/supp/supp_figures/tes/MA0600.1.png]

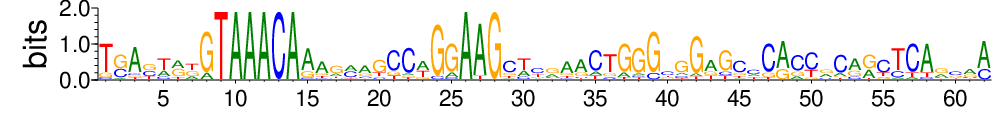

Supplement: btad378_Supplementary_Data [file btad378_supplementary_data.zip › supp_updated_chu/supp/supp_figures/tes/MA0593.1.png]

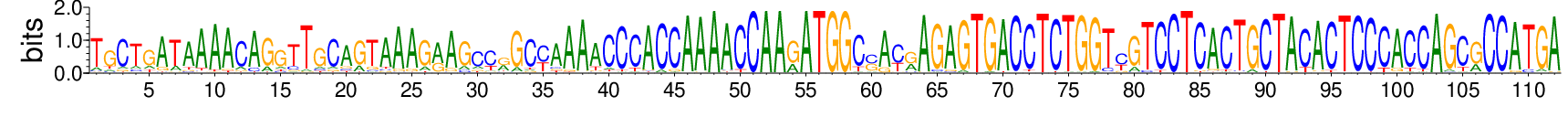

Supplement: btad378_Supplementary_Data [file btad378_supplementary_data.zip › supp_updated_chu/supp/supp_figures/tes/MA0095.2.png]

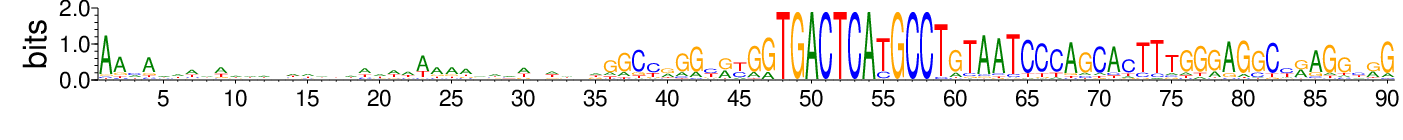

Supplement: btad378_Supplementary_Data [file btad378_supplementary_data.zip › supp_updated_chu/supp/supp_figures/tes/MA0491.1.png]

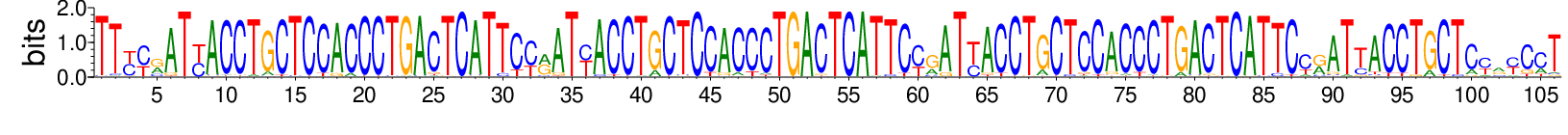

Supplement: btad378_Supplementary_Data [file btad378_supplementary_data.zip › supp_updated_chu/supp/supp_figures/tes/MA0462.1.png]

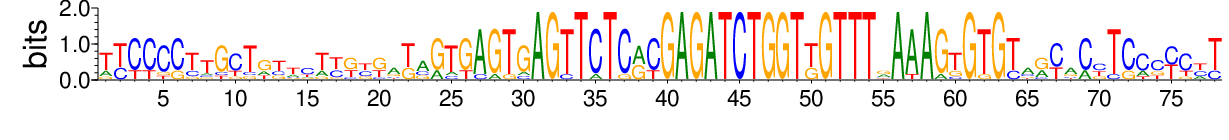

Supplement: btad378_Supplementary_Data [file btad378_supplementary_data.zip › supp_updated_chu/supp/supp_figures/tes/MA0527.1.png]

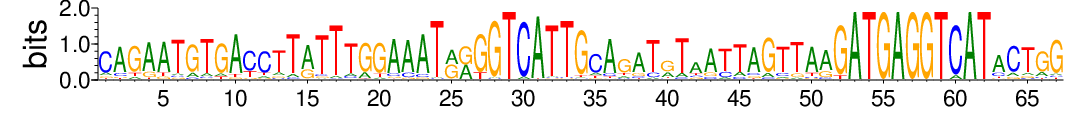

Supplement: btad378_Supplementary_Data [file btad378_supplementary_data.zip › supp_updated_chu/supp/supp_figures/tes/MA0488.1.png]

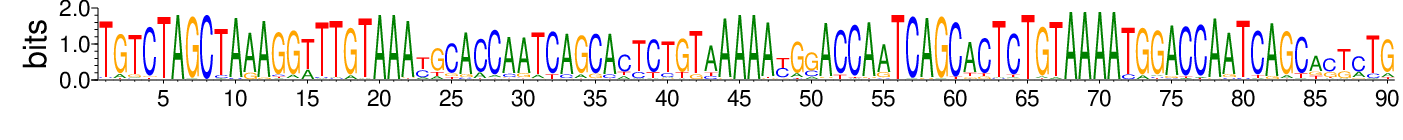

Supplement: btad378_Supplementary_Data [file btad378_supplementary_data.zip › supp_updated_chu/supp/supp_figures/tes/MA0496.1.png]

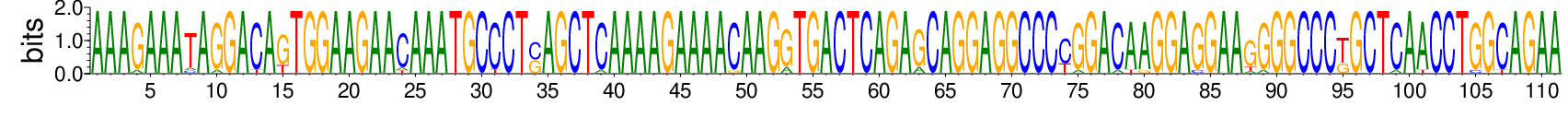

Supplement: btad378_Supplementary_Data [file btad378_supplementary_data.zip › supp_updated_chu/supp/supp_figures/tes/MA0478.1.png]

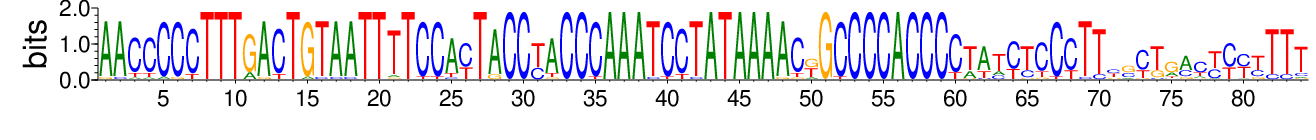

Supplement: btad378_Supplementary_Data [file btad378_supplementary_data.zip › supp_updated_chu/supp/supp_figures/tes/MA0599.1.png]

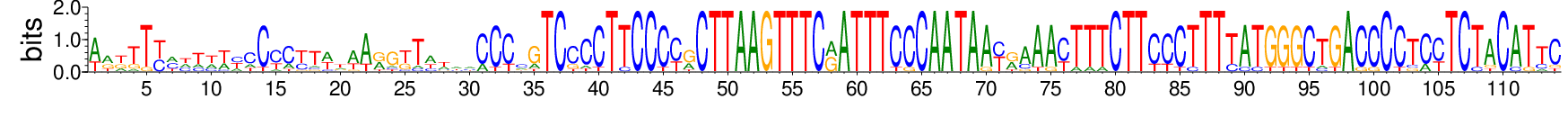

Supplement: btad378_Supplementary_Data [file btad378_supplementary_data.zip › supp_updated_chu/supp/supp_figures/tes/MA0517.1.png]

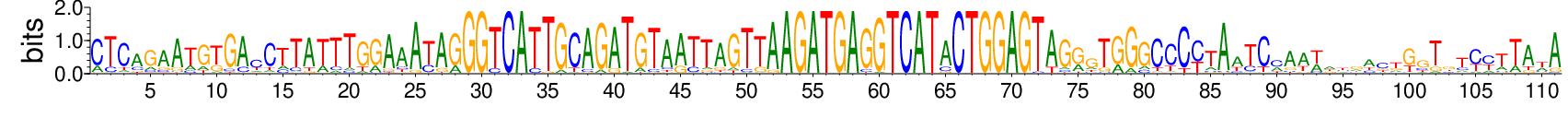

Supplement: btad378_Supplementary_Data [file btad378_supplementary_data.zip › supp_updated_chu/supp/supp_figures/tes/MA0492.1.png]

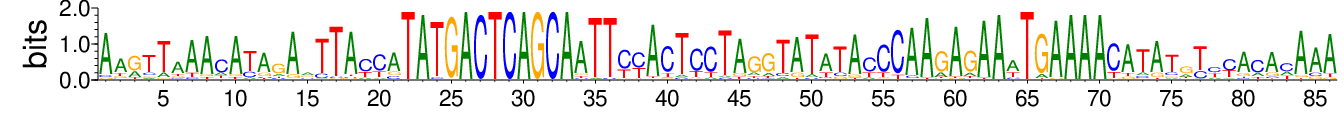

Supplement: btad378_Supplementary_Data [file btad378_supplementary_data.zip › supp_updated_chu/supp/supp_figures/tes/MA0495.1.png]

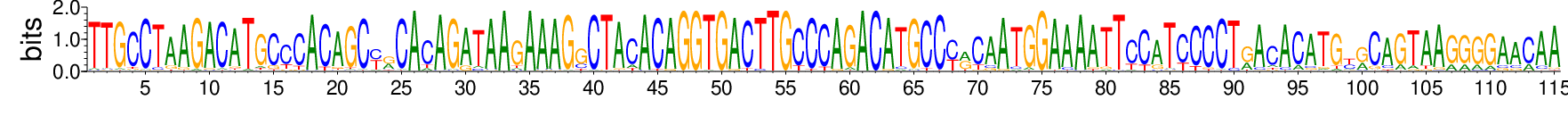

Supplement: btad378_Supplementary_Data [file btad378_supplementary_data.zip › supp_updated_chu/supp/supp_figures/tes/MA0106.2.png]

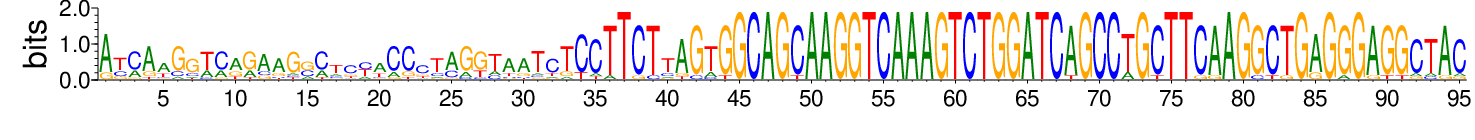

Supplement: btad378_Supplementary_Data [file btad378_supplementary_data.zip › supp_updated_chu/supp/supp_figures/tes/MA0505.1.png]

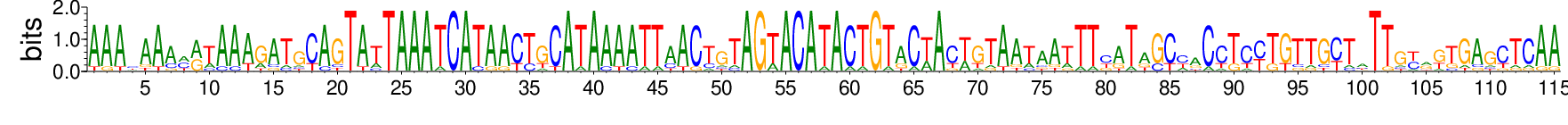

Supplement: btad378_Supplementary_Data [file btad378_supplementary_data.zip › supp_updated_chu/supp/supp_figures/tes/MA0007.2.png]

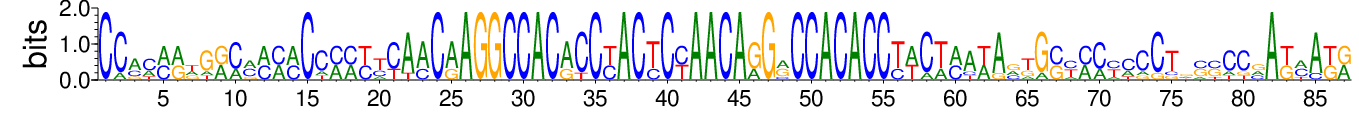

Supplement: btad378_Supplementary_Data [file btad378_supplementary_data.zip › supp_updated_chu/supp/supp_figures/tes/MA0493.1.png]

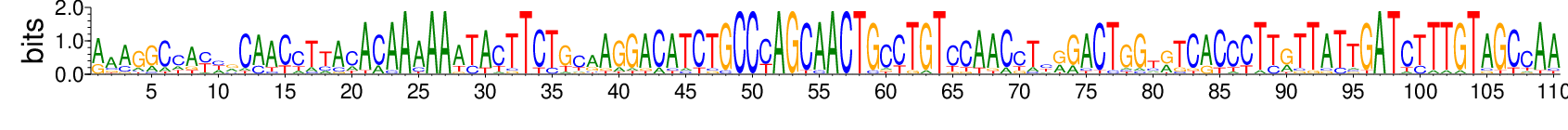

Supplement: btad378_Supplementary_Data [file btad378_supplementary_data.zip › supp_updated_chu/supp/supp_figures/tes/MA0510.1.png]

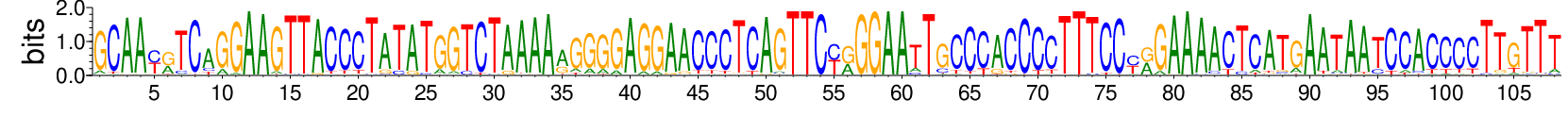

Supplement: btad378_Supplementary_Data [file btad378_supplementary_data.zip › supp_updated_chu/supp/supp_figures/tes/MA0137.3.png]

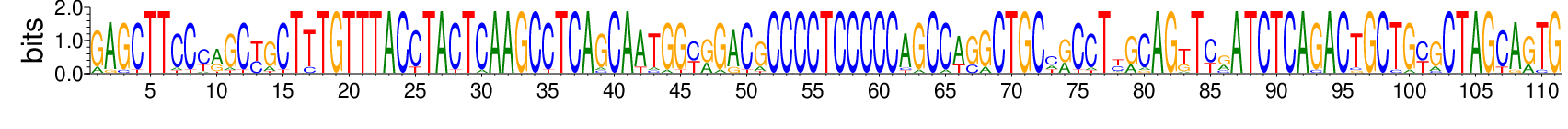

Supplement: btad378_Supplementary_Data [file btad378_supplementary_data.zip › supp_updated_chu/supp/supp_figures/tes/MA0079.1.png]

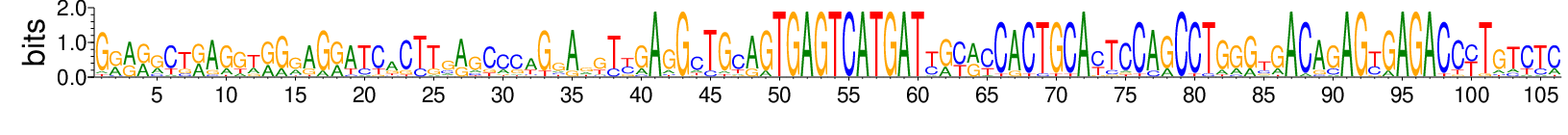

Supplement: btad378_Supplementary_Data [file btad378_supplementary_data.zip › supp_updated_chu/supp/supp_figures/tes/MA0490.1.png]

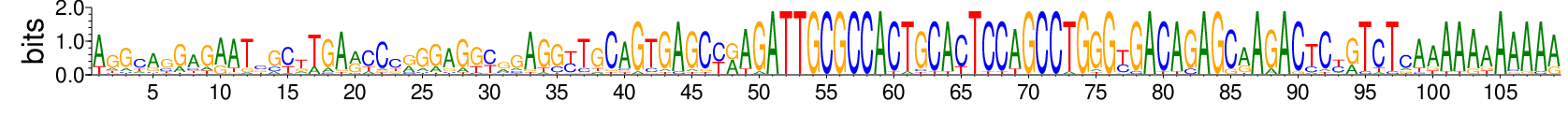

Supplement: btad378_Supplementary_Data [file btad378_supplementary_data.zip › supp_updated_chu/supp/supp_figures/tes/MA0102.3.png]

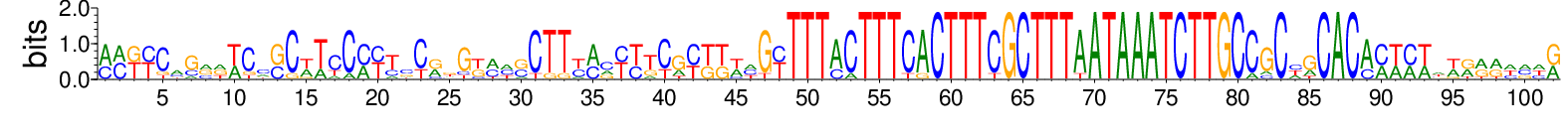

Supplement: btad378_Supplementary_Data [file btad378_supplementary_data.zip › supp_updated_chu/supp/supp_figures/tes/MA0050.2.png]

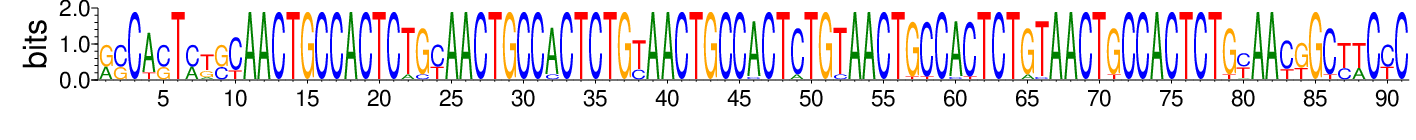

Supplement: btad378_Supplementary_Data [file btad378_supplementary_data.zip › supp_updated_chu/supp/supp_figures/tes/MA0100.2.png]

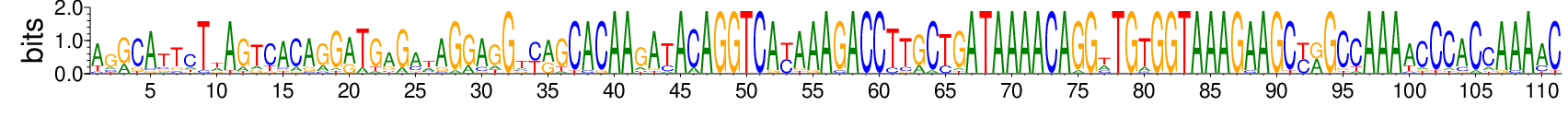

Supplement: btad378_Supplementary_Data [file btad378_supplementary_data.zip › supp_updated_chu/supp/supp_figures/tes/MA0258.2.png]

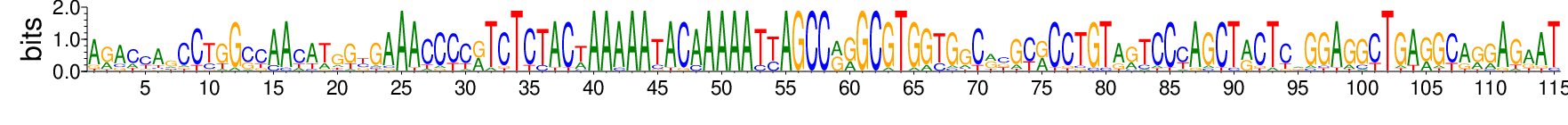

Supplement: btad378_Supplementary_Data [file btad378_supplementary_data.zip › supp_updated_chu/supp/supp_figures/tes/MA0014.2.png]
